# Atrial fibrosis identification with unipolar electrogram eigenvalue distribution analysis in multi-electrode arrays

**DOI:** 10.1007/s11517-022-02648-3

**Published:** 2022-09-13

**Authors:** Jennifer Riccio, Alejandro Alcaine, Sara Rocher, Laura Martinez-Mateu, Javier Saiz, Eric Invers-Rubio, Maria S. Guillem, Juan Pablo Martínez, Pablo Laguna

**Affiliations:** 1 BSICoS Group, Aragón Institute of Engineering Research (I3A), University of Zaragoza, Zaragoza, Spain; 2 CoMBA & BSICoS Groups, Facultad de Ciencias de la Salud, Universidad San Jorge, Zaragoza, Spain; 3 Centro de Investigación e Innovación en Ingeniería, Universitat Politècnica de València, Valencia, Spain; 4 Departamento de Teoría de la Señal y Comunicaciones, Sistemas Telemáticos y Computación, Universidad Rey Juan Carlos, Madrid, Spain; 5 IDIBAPS Institute, Hospital Clínic, Barcelona, Spain; 6 ITACA Institute, Universitat Politècnica de València, València, Spain; 7 Centro de Investigación Biomédica en Red en Bioingeniería, Biomateriales y Nanomedicina (CIBER-BBN), Zaragoza, Spain

**Keywords:** Atrial fibrosis, Atrial fibrillation (AF), Bipolar electrograms (b-EGMs), Eigenvalue dominance ratio (EIGDR), Unipolar electrograms (u-EGMs)

## Abstract

**Abstract:**

Atrial fibrosis plays a key role in the initiation and progression of atrial fibrillation (AF). Atrial fibrosis is typically identified by a peak-to-peak amplitude of bipolar electrograms (b-EGMs) lower than 0.5 mV, which may be considered as ablation targets. Nevertheless, this approach disregards signal spatiotemporal information and b-EGM sensitivity to catheter orientation. To overcome these limitations, we propose the dominant-to-remaining eigenvalue dominance ratio (EIGDR) of unipolar electrograms (u-EGMs) within neighbor electrode cliques as a waveform dispersion measure, hypothesizing that it is correlated with the presence of fibrosis. A simulated 2D tissue with a fibrosis patch was used for validation. We computed EIGDR maps from both original and time-aligned u-EGMs, denoted as $\mathcal {R}$ and $\mathcal{R}^{\mathcal{A}}$, respectively, also mapping the gain in eigenvalue concentration obtained by the alignment, $\Delta \mathcal{R}^{\mathcal{A}}$. The performance of each map in detecting fibrosis was evaluated in scenarios including noise and variable electrode-tissue distance. Best results were achieved by $\mathcal{R}^{\mathcal{A}}$, reaching 94% detection accuracy, versus the 86% of b-EGMs voltage maps. The proposed strategy was also tested in real u-EGMs from fibrotic and non-fibrotic areas over 3D electroanatomical maps, supporting the ability of the EIGDRs as fibrosis markers, encouraging further studies to confirm their translation to clinical settings.

**Graphical Abstract:**

Upper panels: map of $\mathcal {R}^{\mathcal {A}}$ from 3×3 cliques for Ψ= 0^∘^ and bipolar voltage map *V*^*b*-*m*^, performed assuming a variable electrode-to-tissue distance and noisy u-EGMs (noise level *σ*_*v*_ = 46.4 *μV* ). Lower panels: detected fibrotic areas (brown), using the thresholds that maximize detection accuracy of each map 
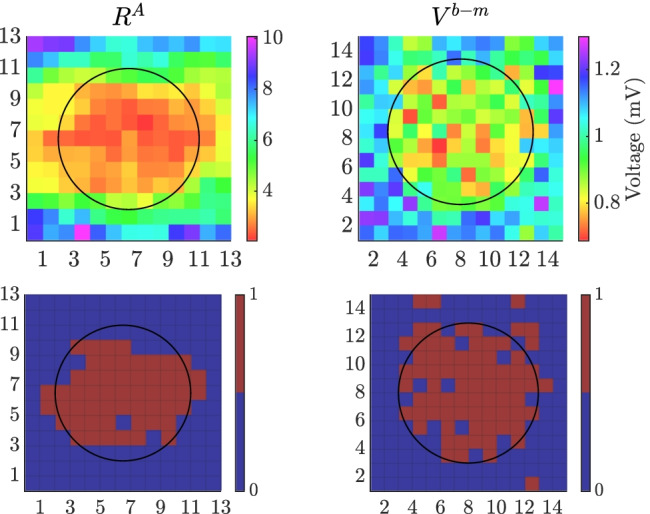

## Introduction

Atrial fibrosis represents a structural anomaly of the atrial myocardium [1]. It is characterized by an altered extracellular matrix activity caused by fibroblasts [2], which alters the electrical conduction and excitability of the tissue. Fibroblasts activation and proliferation, as well as their secretion of extracellular matrix proteins, such as collagen, characterize fibrotic tissue [3]. This structural remodeling mainly occurs during the reparative process to replace damaged myocardial parenchyma [4]. In addition to this replacement process, others, as reactive fibrosis to a trigger as inflammation, have been recognized as responsible for fibrosis. This makes detection of fibrotic tissue even more difficult and suggests the need for more specific imaging tools and markers to detect and quantify fibrosis [5].

Atrial fibrosis has been observed to be correlated to atrial fibrillation (AF) [6]. Despite the fact that AF represents the most common cardiac arrhythmia, its trigger mechanisms are not yet fully understood [7] and its causal relationship with atrial fibrosis is still challenging [1]. On the one hand, fibrosis-induced remodeling creates a substrate promoting AF [1, 6]; on the other hand, fibrosis can occur as a result of the electrical [1] as well as structural [4] atrial remodeling found in AF.

Atrial fibrosis is electrophysiologically characterized by low intracardiac electrograms (EGMs) amplitudes and conduction velocities [8], which may be measured with electroanatomical mapping (EAM) systems [9]. These allow displaying 3D voltage and activation time maps over a reconstruction of cardiac chambers anatomy and visualizing catheter position, so as to guide ablation procedures and treat arrhythmias with minimum radiation exposition [10]. Based on several studies, consensus exists on the choice of 0.5 mV as the threshold value of bipolar EGMs (b-EGMs) peak-to-peak amplitude to discriminate fibrotic areas in the atrium during sinus rhythm [11]. However, this procedure brings along several limitations. First of all, a peak-to-peak voltage measure disregards morphological and temporal information contained in the signal. Nevertheless, this reflects the possible presence of underlying abnormalities in the atria [12]. Secondly, bipolar voltage mapping may be influenced by other technical factors not related to the substrate, including the relative orientation between the recording electrode pair and wavefront propagation direction, electrode size, inter-electrode distance and b-EGMs filtering [12]. Bipolar voltage is also affected by the tissue-electrode contact, whose maintenance may be challenging in anatomically difficult sites (e.g., the pulmonary veins). Third, the definition of low-voltage areas has not been subjected to a standardization procedure and the voltage threshold has never received an histological validation [12].

In recent years, more attention has been paid to the role of fibrotic tissue on the initiation and perpetuation of AF than on its effects on the morphology of the EGMs [13]. In this sense, not many intracardiac signal processing methods based on EGM features have been proposed to detect fibrosis. Some studies have revealed the relationship of EGM morphology and tissue alterations, including ablation lesion formation [14]. Others have introduced a method to characterize the different fibrotic textures based on EGM fractionation due to the incidence of wavefront direction [15]. All these works have used *in silico* experiments for validation.

In this paper, we hypothesize that the waveform dispersion of neighbor unipolar EGMs (u-EGMs) is correlated with the presence of atrial fibrosis. Therefore, the aim of the work is to propose eigenvalue-based indices of waveform dispersion to identify fibrotic areas. They take into account the spatiotemporal relations of the u-EGM waveforms and overcome the limitations of the use of b-EGM voltage thresholding for the detection of fibrosis. Resulting maps, called eigenvalue dominance ratio (EIGDR) maps, were computed with a simulation setup, using two sizes of nearby electrode arrangements, referred as $\textit{cliques}$ (2$\times$2 and 3$\times$3 electrodes), and three catheter orientations with respect to the tissue preferential direction (0$^{\circ }$, 30$^{\circ }$ and 45$^{\circ }$). The ability of each map was evaluated in detecting a fibrosis patch included in the simulated tissue. As a proof of concept, EIGDR values were also computed in clinical u-EGMs from patients, in four- and five-electrode cliques. They were correlated with the presence/absence of fibrosis based on late gadolinium enhancement-magnetic resonance imaging.

This paper is organized as follows: Section 2 presents the methodology and the datasets used for its validation. Section 3 contains the obtained results, whereas Sections 4, 5 and 6 include the discussion, limitations (with references to challenges and future perspectives) and conclusions of the work, respectively.

## Methods

### Atrial model

**Fig. 1:**
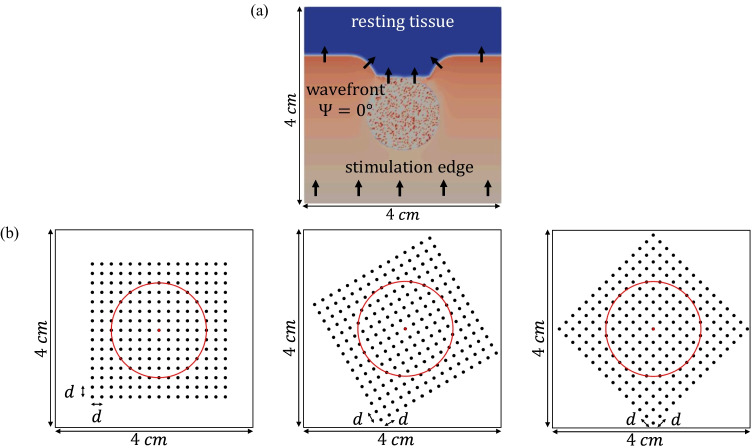
(a) Activation distribution at a particular time instant over the 2D tissue used in this work, including the fibrotic patch. Black arrows indicate propagation wavefront direction. (b) The three MEA orientations with respect to the tissue considered in this study: $\Psi = 0^{\circ }$ (leftmost), $\Psi = 30^{\circ }$ (middle) and $\Psi = 45^{\circ }$ (rightmost), where the red circle encompasses the fibrotic tissue area. It should be noted that representation in (a) refers to the relative orientation between tissue and propagation direction corresponding to $\Psi = 0^{\circ }$

We simulated an atrial tissue slice of 4$\times$4 cm by dividing it into adjacent square elements whose centers were separated 0.1 mm. Within the tissue slice, a circular patch with a diameter of 2 cm was defined, whose center was at the center of the 2D tissue, as shown in Fig. 1(a). Inside the patch, a fully transmural (from endo- to epicardial layer) pattern of diffuse fibrosis was randomly defined following a uniform distribution. The Maleckar model for myofibroblasts [16] was assigned to 20% of the nodes within the circular area. Although the percentage of atrial fibrosis is very patient-dependent, the density of 20% represents the threshold value between stage II and stage III according to the Utah classification [17] and therefore considered a realistic percentage for this study.

The cell model assigned to all the non-fibrotic nodes was a variant of the Courtemanche myocyte model [18] accounting for the atrial electrophysiological characteristics experimentally observed in the left atrium (LA) and the persistent AF (cAF) induced remodeling [19]. This electrical remodeling was introduced through the variation of the maximum conductances of the transient outward potassium current $(I_{to})$, the L-Type calcium current $(I_{CaL})$, the inward rectifier potassium current $(I_{K1})$, the ultrarapid outward potassium current $(I_{Kur})$ and the slow delayed rectifier potassium current $(I_{Ks})$ (see Table 1), as in previous computational studies [19, 20]. Additionally, the diffusion tensor was adjusted to reproduce the conduction velocity in the LA, and was reduced by 30% in all elements of the patch with at least one fibroblast node [21], similarly to what was performed in [22].

**Fig. 2:**
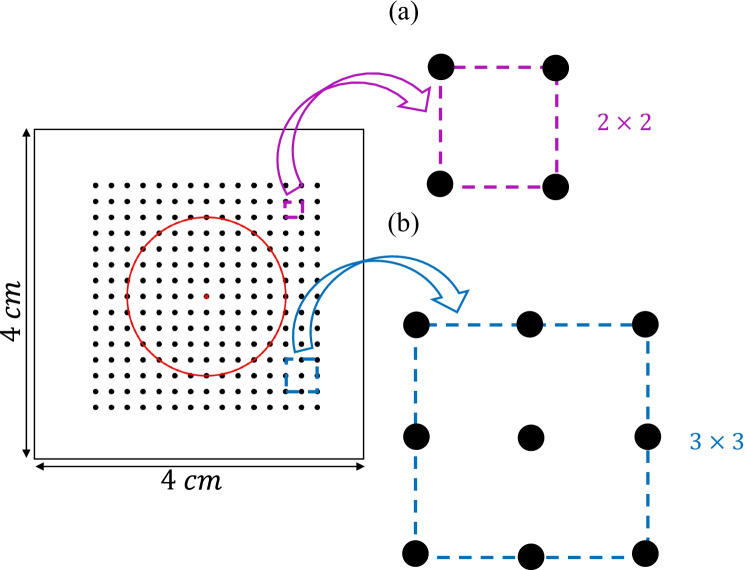
Arrangements of four (a) and nine (b) electrodes (2$\times$2 and 3$\times$3 cliques, respectively) from the MEA

Simulations were run in ELVIRA software [23]. The monodomain formulation was solved using the operator splitting numerical scheme with a constant time step $\delta t$ = 0.01 ms and a spatial resolution $\delta x$ = 0.1 mm. Recording electrodes were distributed over the simulated anatomy mimicking a *L* $\times$ *L* high-density multi-electrode array (MEA) (*L* = 15), where the inter-electrode distance was *d* = 2 mm. The simulated electrode grid was located so that its central electrode corresponds to the center of both the tissue slice and the fibrotic patch, and was rotated by an angle $\Psi$, $\Psi \in \{0^{\circ }, 30^{\circ }, 45^{\circ }\}$, with respect to the tissue fiber direction. Fig. 1(b) shows the three MEA orientations over the simulated tissue geometry. Two generic cliques of different sizes were considered in this work as depicted in Fig. 2(a) and (b), forming four and nine-electrode square arrangements, respectively. Each clique location is referred to the lower left electrode, indexed as (*i*, *j*), within the MEA. The rest of electrodes in the clique are numbered from left to right and bottom to top, thus corresponding to locations $(i+\Delta _{i},j+\Delta _{j})$, where $\Delta _{i},\Delta _{j} \in \{0,1\}$, ($i,j\in\{1,\cdots,14\}$) for the $2\times 2$, and $\Delta _{i},\Delta _{j} \in \{0,1,2\}$, ($i,j \in \{1,\cdots ,13\}$) for the $3\times 3$ arrangements, respectively.

**Table 1:** Variation of the maximum conductances *g* for several ionic channels used to reproduce atrial electrical remodeling under persistent AF (cAF) conditions, accordingly to experimental studies reported in literature. As a comparison, *g* values have also been reported in control conditions

	$g_{to}$	$g_{CaL}$	$g_{K1}$	$g_{Kur}$	$g_{Ks}$
Control	1.00	1.00	1.00	1.00	1.00
cAF	0.25	0.35	2.00	0.55	2.00
References	[24]	[25, 26]	[27–29]	[24]	[24]

### Synthetic signals

**Fig. 3:**
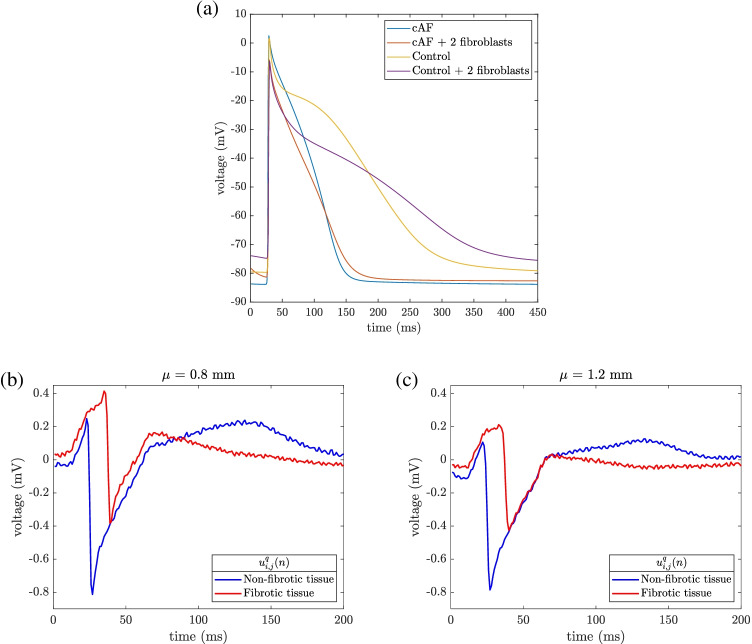
Upper panel (a): action potentials (APs) in persistent atrial fibrillation (cAF), registered in two different nodes from the simulation mesh: in a cardiomyocyte outside the fibrotic patch (light blue line) and in a cardiomyocyte inside the fibrotic patch coupled with two fibroblasts (orange line). In order to show the effect of the applied electrical remodeling, APs were also shown in control conditions, from different simulations not including electrophysiological remodeling and not used in this work (yellow and purple lines, for uncoupled and coupled cardiomyocytes, respectively). Lower panel: Noisy unipolar EGMs $u^{q}_{i,j}(n)$ ($\sigma _{v} = 46.4 \ \mu V$) recorded in non-fibrotic, $(i,j) = (3,3)$, and fibrotic $(i,j) = (8,7)$ tissue areas (blue and red line, respectively) when $\Psi =0^{\circ }$, at electrode-to-tissue distances of ${\mu _{3,3}=\mu _{8,7}= 0.8}$ mm (b) and ${\mu _{3,3}=\mu _{8,7}=1.2}$ mm (c)

Unipolar EGMs, $u_{i,j}(n)$, were calculated as originated by the passage of the propagation wavefront by electrodes located at sites (*i*, *j*) of the MEA. We computed them in a volumetric tissue-blood model, assuming a temporal resolution of 1 ms, as done in [30]. First, extracellular potentials were obtained by an approximation of the bidomain formulation, considering the tissue immersed in a non-conductive bath. In order to reproduce fibrosis effects, inside the simulated fibrotic patch the single cardiomyocyte under cAF conditions was coupled with a randomly variable number of fibroblasts within the patch. Fig. 3(a) shows the action potentials registered in two different cardiomyocytes from the mesh, one outside the fibrotic patch and the other inside the fibrotic patch, where it is coupled with two fibroblasts. Electrical remodeling induced by cAF produces a 55% reduction in duration measured at 90% repolarization (248 vs. 111 ms), in concordance with experimental data [29]. Fibroblast coupling with cAF cardiomyocytes makes resting potential less negative (83 vs. 78 mV) and elongates the duration measured at 90% repolarization (111 vs. 120 ms). Then, u-EGMs were solved in the entire domain by the governing equations for a solid volume conductor and its boundary conditions at the tissue-blood interface.

In order to take into account a realistic scenario, where the distance from the tissue may not be constant through the electrodes in the clique, we considered that the electrode-tissue distance, $\mu _{i,j}$, varies following a normal distribution with mean $\overline{\mu }$ = 1 mm and standard deviation $\sigma _{\mu }$ = 0.1 mm. Two thousand different random configurations were simulated, where the distance of each electrode to the tissue was randomly and independently chosen following that distribution.

Synthetic u-EGMs were computed with sampling frequency of 1 kHz, duration of 0.5 s (${N=500}$ samples), and including one single activation (depolarization plus repolarization).

Simulated signals were corrupted with noise excerpts obtained from real u-EGMs, as previously done in [8]. Two thousand different noise segments were extracted from u-EGMs recorded with a multi-electrode PentaRay^®^ catheter (*Biosense-Webster, Inc., Diamond Bar, CA, USA*) at intervals with no recorded EGMs. All noise segments were normalized to have standard deviations ${\sigma _{v}\in \{0.0, 5.8, 11.6, 23.2, 46.4\} \ \mu V}$, corresponding to peak-to-peak amplitudes ${\overline{V}_{pp,v}\in \{0.0, 24.2, 48.4, 96.7, 193.5\} \ \mu V}$. They are concordant with observed average power in unipolar recordings [31] and guarantee a homogeneous power level. Different realizations, indexed by ${q\in \{1,\cdots , 2000\}}$, of this recorded noise were randomly added to each one of the two thousand realizations of the simulated u-EGMs $u_{i,j}(n)$ computed with a variable electrode-tissue distance within the MEA, generating as a result noisy unipolar signals $u^{q}_{i,j}(n)$, ${i,j\in \{1,\cdots ,15 \}}$, ${q\in \{1,\cdots ,2000\}}$. Noisy u-EGMs examples, in fibrotic and non-fibrotic tissue zones, are shown in Fig. 3(b) and (c), at two different electrode-tissue distances, respectively, with noise level corresponding to $\sigma _{v} = 46.4 \ \mu V$.

### Clinical data

**Fig. 4:**
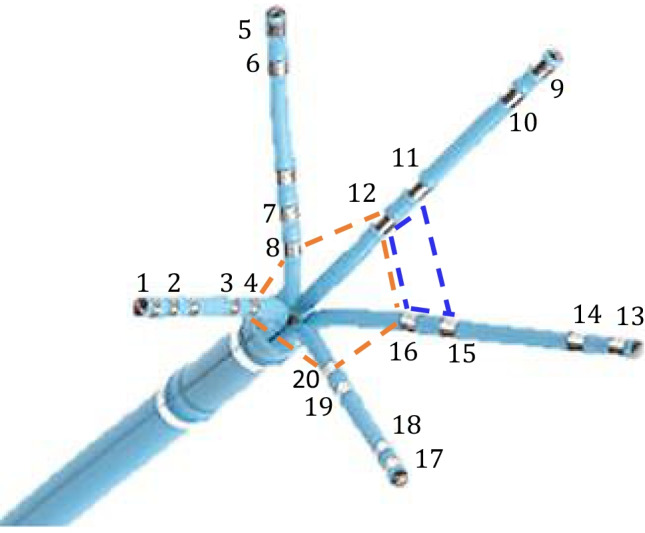
The PentaRay^®^ catheter, where the 20 poles are highlighted. Two of the clique distributions considered around each catheter mapping point, with four (dashed blue line) and five (dashed orange line) electrodes, are also pointed out. This image was modified from the Biosense Webster catalog

Intracavitary u-EGMs recorded during sinus rhythm with a PentaRay^®^ catheter (*Biosense-Webster, Inc., Diamond Bar, CA, USA*) were used to evaluate performance of EIGDR approach with real signals. Clinical data were obtained from a patient with cAF, a slightly dilated LA (26 cm$^{2}$), a left ventricular ejection fraction of 58% and treated with anticoagulation and flecainide, registered at the Hospital Clínic, Barcelona, Spain. The data acquisition protocol was reviewed and approved by the Hospital Clinic Ethical Committee (Ethics approval number: HCB/2019/0881). The patient was informed and signed the consent form. A total of 758 mapping points (or catheter sites) were acquired at the anterior, posterior, lateral and septal wall, as well as the left atrial appendage and the pulmonary veins of the LA (left atrial regions) using the CARTO^®^ 3 EAM system (*Biosense-Webster, Inc., Diamond Bar, CA, USA*), so as to reconstruct a real-time 3D anatomical map before the ablation procedure. Signals were acquired from the 20 poles distributed among the five branches of the catheter, Fig. 4, characterized by consecutive inter-electrode spacings *d*, at each branch, of 2, 6, and 2 mm, resulting in 20 u-EGMs associated at each mapping point. In order to evaluate performance of EIGDR-based markers, 38 catheter positions were selected by an operator-dependent visual approach. Specifically, we visually identified and manually annotated nineteen points clearly assignable to fibrotic and another nineteen points to non-fibrotic areas on the anatomic map. In order to guide this decision, the corresponding magnetic resonance image (MRI) was used as reference.

Three of the catheter positions selected at fibrosis showed poor electrode-tissue contact for one or more splines of the catheter. In addition, mapping points located at borderline areas between fibrotic and healthy tissues over the MRI and/or the bipolar voltage map, which showed bipolar amplitudes unhealthy (< 0.1 mV) or poor healthy (< 0.5 mV), have been excluded from the analysis.

**Fig. 5:**
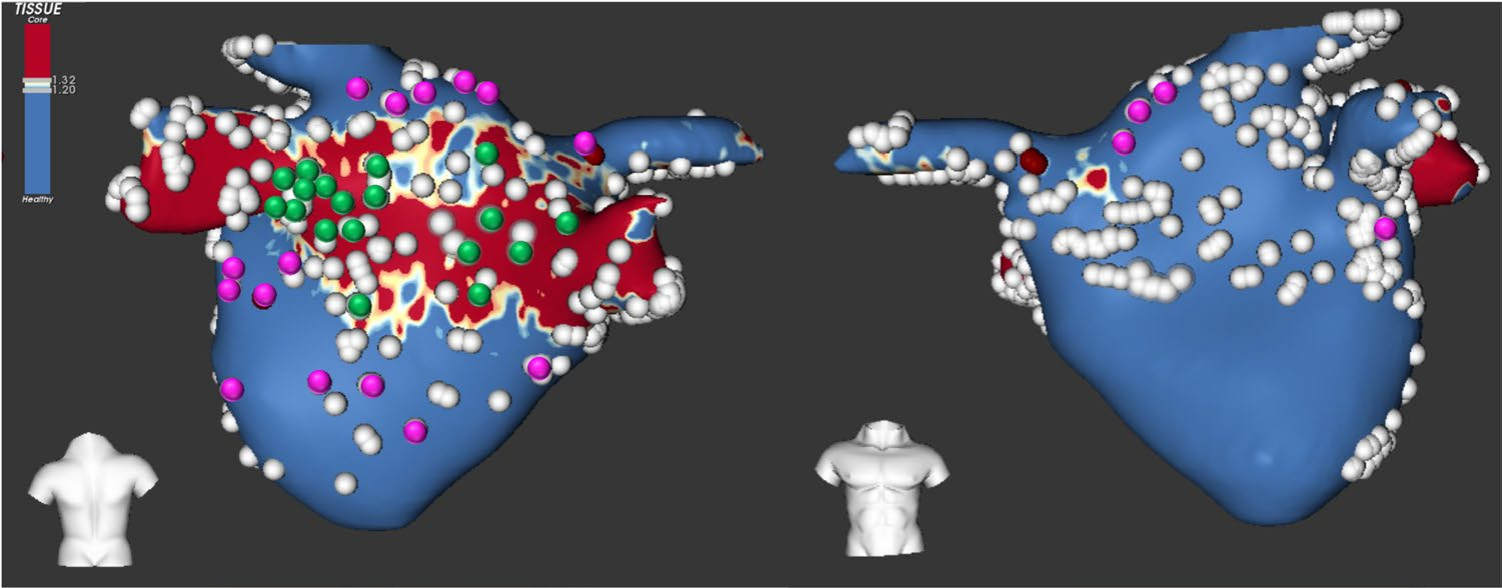
Posteroanterior (left) and anteroposterior (right) views of color-coded 3D mesh of MRI (showing dense fibrosis in red and healthy tissue in blue) generated by ADAS 3D co-registered with all EAM mapping points provided by CARTO 3 (gray). The 38 mapping points selected over fibrotic and non-fibrotic areas to compute EIGDR and bipolar indices are highlighted in green and magenta, respectively

EAM data and MRI were co-registered with the ADAS 3D Medical imaging software (*ADAS-3D, Barcelona, Spain*), as shown in Fig. 5. That co-register was performed by manually selecting some landmarks (between six and ten) in specific areas (such as the pulmonary veins and the atrial appendage) of the meshes. In order to determine how pathological the tissue is, the methodology described in [32] was used. Following the image intensity ratio (IIR) based thresholding, a color-coded 3D mesh was automatically generated, showing in blue the healthy tissue (IIR<1.2) and in red the dense fibrosis (IIR>1.32) (Fig. 5).

**Fig. 6:**
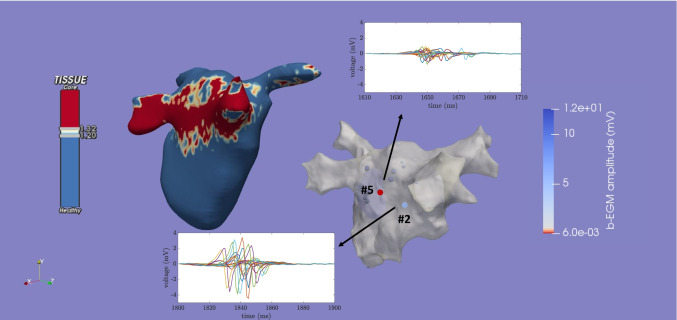
3D reconstruction of the LA geometry (gray mesh) and corresponding co-registered MRI, showing the different regional distribution patterns of gadolinium (red areas: latest contrast enhancement, blue areas: absence of latest contrast enhancement). In the geometrical mesh, two of the mapping points acquired and considered in the analysis (point #5 at fibrosis, point #2 at non-fibrosis) are marked and color-coded according to their corresponding bipolar peak-to-peak amplitude. For each of them, the atrial activation windows extracted from the twenty filtered u-EGMs recorded with the PentaRay^®^ catheter are also displayed. Note that not all displayed u-EGMs recorded at a particular catheter site belong to a clique, see Section 2.3, and therefore affect the EIGDR indices and bipolar amplitude computations

The u-EGMs were acquired with a sampling frequency of 1 kHz during 2.5 s (2500 samples) and include several activations, each containing the atrial depolarization, followed by the ventricular depolarization and repolarization. Unipolar signals were then high-pass filtered with 30 Hz cutoff frequency, using a third order Butterworth infinite impulse response filter, so as to reduce artefacts and emphasize more rapid components. An example of filtered u-EGMs at two different mapping points marked on the anatomical 3D mesh of the atrium is presented in Fig. 6, where the atrial activation was plotted. At each catheter position, u-EGMs were considered in the following five four-electrode cliques, according to the pole numbering in Fig. 4: (3,4,7,8), (7,8,11,12), (11,12,15,16), (15,16,19,20), (19,20,3,4) and the five-electrode clique (4,8,12,16,20), for their smaller inter-electrode spacing. As example, cliques (3,4,7,8) and (4,8,12,16,20) are depicted in Fig. 4.

### Eigenvalue analysis

Unipolar signals in the clique can be compactly represented by the following $N \times K$ matrix, $K\in \{4,9\}$, as in [33]:

1 $$\begin{aligned} \mathbf {U} = \left[ \begin{array}{lll} \mathbf {u}_{1} &{} \cdots &{} \mathbf {u}_{K} \\ \end{array}\right] , \end{aligned}$$

where the *k*-th column, $\mathbf {u}_{k}$, contains the samples of the unipolar signal $u_{k}(n)$, modeled later in Section 2.6:

2 $$\begin{aligned} \mathbf {u}_{k} = \left[ \begin{array}{lll} {u_{k}(0)}&\cdots&{u_{k}(N-1)} \end{array}\right] ^{T}. \end{aligned}$$

We propose and assess EIGDR values from the clique u-EGMs in (1) to detect fibrosis. The *N* eigenvalues $\{\lambda _{1}, \cdots , \lambda _{N}\}$ of the *N* $\times$ *N* intra-signal correlation matrix $\mathbf {R}_{u} = E[ \mathbf {u}_{k} \mathbf {u}_{k}^{T} ]$ were obtained from the following correlation matrix estimate within each clique:

3 $$\begin{aligned} \hat{ \mathbf {R}}_{u} = \frac{1}{K}\mathbf {U}\mathbf {U}^{T}. \end{aligned}$$

The matrix $\mathbf {R}_{u}$ is the intra-signal sample correlation matrix, whose eigenvalues reflect the degree of morphological variability among the signals in the clique.

For each clique, the ratio $\mathcal {R}$ of the largest (i.e. *dominant*) eigenvalue $\lambda _{1}$ and the remaining ones was estimated:

4 $$\begin{aligned} {\mathcal {R}}= \frac{\lambda _{1}}{\sum _{n=2}^{N} \lambda _{n}}. \end{aligned}$$

By comparing the first eigenvalue to the sum of the others, we are able to quantify the u-EGM energy percentage which can be explained by the shape of the first eigenvector. The ratio $\mathcal {R}$ would be much higher than one if all u-EGMs were essentially identical to each other and all the waveforms can be explained with just the shape of the first eigenvector (i.e., low morphological variability). On the contrary, when waveform dispersion appears, the eigenvalues from $\lambda _{2}$ to $\lambda _{N}$ become higher, thus reducing the ratio $\mathcal {R}$.

### Wave alignment

Eigenvalues of $\hat{ \mathbf {R}}_{u}$ were computed from the original u-EGMs and after intra-clique time alignment, proposed to compensate the effect that different activation wavefront arrival times have on the EIGDR. In this case, unipolar signals were aligned according to Woody’s iterative procedure [34, 35]:

1. at first *l*-th iteration, ${l=0}$, the relative time delay ${\hat{\tau }_{k,0}}$ between each unipolar signal $u_{k}(n)$ and the u-EGM with the highest peak-to-peak amplitude within the clique $u_{max}(n)$ (assumed as initial reference signal) was estimated by maximizing their cross-correlation:
  5 $$\begin{aligned} {\hat{\tau }_{k,0}} = \arg \max _{\tau } \sum _{n=0}^{N-1} \; u_{k}\left( n-\tau \right) \; u_{max}(n); \end{aligned}$$
2. from the relative time delays, the average of the shifted signals $u_{k}(n-\hat{\tau }_{k,0})$ within the clique was calculated:
  6 $$\begin{aligned} \bar{u}_{0}(n) = \frac{1}{K}\sum _{k=1}^{K} u_{k}\left( n-\hat{\tau }_{k,0}\right) ; \end{aligned}$$
3. in each *l*-th iteration, ${l>0}$, the cross-correlation between each $u_{k}(n)$ and the average signal obtained in the previous iteration $\bar{u}_{l-1}(n)$ (assumed as updated reference signal) is maximized to find the updated relative time delays ${\hat{\tau }_{k,l}}$:
  7 $$\begin{aligned} {\hat{\tau }_{k,l}} = \arg \max _{\tau } \sum _{n=0}^{N-1} \; u_{k}\left( n-{\tau }\right) \; \bar{u}_{l-1}\left( n\right) . \end{aligned}$$
  This process is repeated iteratively until the delay estimates no longer change.

Eigenvalues of the intra-signal correlation matrix of the aligned u-EGMs were calculated in the same way as for their non-aligned version, thus leading to the formulation of the ratio $\mathcal{R}^{\mathcal{A}}$ (where the upper index denotes that the ratio comes from aligned u-EGMs within the clique) analogous to (4).

### Unipolar signal modeling

The u-EGM signals within a clique located at (*i*, *j*) position within the MEA are indexed as $u_{k}(n)$, ${k\in \{1,\cdots , K \}}$, ${K\in \{4,9\}}$. Assuming a plane wave propagation, the different electrodes in a clique are activated at different times, and therefore u-EGMs in the clique $u_{k}(n)$ will be delayed versions among them, plus noise and non-homogeneous components. Therefore, they can be modeled, analogously to [36] for misaligned signal ensembles, as:

8 $$\begin{aligned} u_{k}(n) = \alpha _{k} s(n-\tau _{k})+f_{k}(n)+v_{k}(n) \ \ {\left\{ \begin{array}{ll} k = 1,\cdots ,K\\ n =1,\cdots ,N \end{array}\right. }, \end{aligned}$$

where:

- *s*(*n*) is the u-EGM activation signal component assumed to be space invariant in the case of a plane wave propagation and homogeneous tissue free of fibrosis. Its energy is denoted as $E_{s}$.
- $\tau _{k}$ is the delay of the *k*-th u-EGM $s(n-\tau _{k})$ with respect to a time reference within the clique, introduced later. Delays $\tau _{k}$ are zero-mean and characterized by their variance in the normal tissue $\sigma _{\tau }^{2}$. In fibrotic areas, the reduction in conduction velocity with respect to healthy tissue increases the variance of the $\tau _{k}$ up to $\beta ^{2}\sigma _{\tau }^{2}$, where the factor $\beta$ > 1 in fibrosis and $\beta =$ 1 in non-fibrotic tissue.
- $\alpha _{k}$ is a parameter accounting for u-EGM amplitude reduction between fibrotic, $\alpha _{k}<1$, and healthy, $\alpha _{k}=1$, tissues. It is modeled as a random variable with mean $\overline{\alpha }$ and variance $\sigma _{\alpha }^{2}$ ($\overline{\alpha }=1$, $\sigma _{\alpha }^{2}=0$ in healthy tissue; $\overline{\alpha }<1$, $\sigma _{\alpha }^{2}>0$ in fibrosis).
- $f_{k}(n)$ is a zero-mean fibrotic signal component across the clique with variance ${\sigma _{f}^{2}}$. In healthy tissue ${f_{k}(n)=0}$.
- $v_{k}(n)$ is the zero-mean noise component at the *k*-th u-EGM, with variance ${\sigma _{v}^{2}}$, Gaussian, white and uncorrelated with $\tau _{k}$ and $f_{k}(n)$.

Four different scenarios for each $u_{k}(n)$ were considered in this study, as already proposed in [37], with/without fibrosis and with/without prior alignment. Their approximate theoretical eigenvalues and EIGDR were derived following parallel methodology to that used in [36] for repetitive signal ensemble alignment, as detailed below.

#### Prior alignment with no fibrosis

For perfectly aligned signals without fibrosis, i.e., ${u_{k}(n) = s(n) + v_{k}(n)}$, the intra-signal correlation matrix is given by:

9 $$\begin{aligned} \mathbf {R}_{u} = \mathbf {s}\mathbf {s}^{T} + {\sigma _{v}^{2}} \mathbf {I}, \end{aligned}$$

where $\mathbf {I}$ is the $N \times N$ identity matrix and data vector $\mathbf {s}$, $${\mathbf {s} = \left[ \begin{array}{lll} s(0)&\cdots&s(N-1)\end{array}\right] ^{T}}$$, is easily shown to be proportional to the first eigenvector of $\mathbf {R}_{u}$, whereas the remaining eigenvectors are chosen arbitrarily as long as they are orthogonal to the first. The eigenvalues are given by:

10 $$\begin{aligned} \lambda _{n} = {\left\{ \begin{array}{ll} E_{s} + {\sigma^{2}_{v}} &{} n=1\\ {\sigma ^{2}_{v}} &{} n=2, \cdots , N, \end{array}\right. } \end{aligned}$$

where $E_{s}=\mathbf {s}^{T}\mathbf {s}$ is the signal energy. For real u-EGM, signal energy is much larger than noise energy, ${E_{s}>>N {\sigma _{v}^{2}}}$, and ${N>>1}$, resulting in an EIGDR of:

11 $$\begin{aligned} \mathcal{R}^{\mathcal{A}} \approx \frac{E_{s}}{N{\sigma _{v}^{2}}}. \end{aligned}$$

#### No prior alignment and no fibrosis

We now analyze the case of raw u-EGMs in the clique where misalignment of *s*(*n*) is assumed to be present in each *k*-th u-EGM, ${u_{k}(n)=s(n-\tau _{k})+v_{k}(n)}$. Then, to estimate the eigenvalues we can approximate $u_{k}(n)$, for small $\tau _{k}$, as [36]:

12 $$\begin{aligned} u_{k}(n) \approx s(n)- \tau _{k} s^{\prime }(n) + \frac{1}{2} {\tau _{k}^{2}} s^{\prime \prime }(n) +v_{k}(n), \end{aligned}$$

where $s^{\prime }(n)$ and $s^{\prime \prime }(n)$ denote the first and second derivative of *s*(*n*), respectively. The intra-signal correlation matrix can be expressed as:

13 $$\begin{aligned} \mathbf {R}_{u} \approx \left( \mathbf {s}\mathbf {s}^{T} + \frac{\sigma _{\tau }^{2}}{2} (\mathbf {s}\mathbf {s}^{\prime \prime T}+\mathbf {s}^{\prime \prime }\mathbf {s}^{T})\right) + \sigma _{\tau }^{2} \mathbf {s}^{\prime }\mathbf {s}^{\prime T}+ {\sigma _{v}^{2}} \mathbf {I}, \end{aligned}$$

where $\mathbf {s}^{\prime }$ and $\mathbf {s}^{\prime \prime }$ are the vector counterparts of $s^{\prime }(n)$ and $s^{\prime \prime }(n)$, respectively. It can be shown that the eigenvalues of $\mathbf {R}_{u}$ are approximated by [36]:

14 $$\begin{aligned} \lambda _{n} \approx {\left\{ \begin{array}{ll} E_{s} - \sigma _{\tau }^{2} E_{s^{\prime }} + {\sigma ^{2}_{v}} &{} n=1\\ \sigma _{\tau }^{2} E_{s^{\prime }} + {\sigma ^{2}_{v}} &{} n=2 \\ {{\sigma ^{2}_{v}}} &{} n=3, \cdots , N, \end{array}\right. } \end{aligned}$$

where $E_{s^{\prime }}=\mathbf {s}^{\prime T}\mathbf {s}^{\prime }$ is the derivative signal energy. The resulting EIGDR is approximated by:

15 $$\begin{aligned} \mathcal {R} \approx \frac{E_{s} - \sigma _{\tau }^{2} E_{s^{\prime }}}{\sigma _{\tau }^{2} E_{s^{\prime }} + N{\sigma _{v}^{2}}}. \end{aligned}$$

Note that when $\sigma _{\tau }=0$ (i.e., perfect alignment) this equation becomes equal to (11).

#### Prior alignment and fibrosis

When u-EGMs are first aligned in fibrosis zones, each of them can be modeled as ${u_{k}(n) = \alpha _{k} s(n)+f_{k}(n)+v_{k}(n)}$. The correlation matrix results in:

16 $$\begin{aligned} \mathbf {R}_{u} = \left( \overline{\alpha }^{2}+\sigma _{\alpha }^{2}\right) \mathbf {s}\mathbf {s}^{T} + {\sigma _{v}^{2}} \mathbf {I} + {\sigma _{f}^{2}} \mathbf {I}, \end{aligned}$$

and their corresponding eigenvalues are:

17 $$\begin{aligned} \lambda _{n} \approx {\left\{ \begin{array}{ll} {\left( \overline{\alpha }^{2}+\sigma _{\alpha }^{2}\right) E_{s}+{\sigma _{v}^{2}} + {\sigma _{f}^{2}}} &{} n=1\\ {{\sigma _{v}^{2}} + {\sigma _{f}^{2}}} &{} n=2, \cdots , N, \end{array}\right. } \end{aligned}$$

which lead to:

18 $$\begin{aligned} \mathcal{R}^{\mathcal{A}}_{\mathcal{F}} \approx \frac{ E_{s}}{\frac{N({\sigma _{v}^{2}}+ {\sigma _{f}^{2}})}{\left( \overline{\alpha }^{2}+\sigma _{\alpha }^{2}\right) }}, \end{aligned}$$

where the parameters used have already been introduced at the beginning of this section and upper $\mathcal {A}$ and lower $\mathcal {F}$ indices in $\mathcal{R}_{\mathcal{F}}^{\mathcal{A}}$ denote that the calculations are obtained from intra-clique aligned u-EGMs in fibrotic tissue, respectively.

#### No prior alignment with fibrosis

When raw u-EGMs come from fibrotic areas, each of them can be modeled as ${u_{k}(n) = \alpha _{k} s(n-\tau _{k})+f_{k}(n)+v_{k}(n)}$. In this case, the delay $\tau _{k}$ has larger standard deviation than in non-fibrotic areas, with the extra delay controlled by a multiplicative factor $\beta$, thus resulting in the following correlation matrix:

19 $$\begin{aligned} \mathbf {R}_{u} \approx & (\overline{\alpha }^{2}+\sigma _{\alpha }^{2})\left[ \left( \mathbf {s}\mathbf {s}^{T} + \frac{\beta ^{2}\sigma _{\tau }^{2}}{2} (\mathbf {s}\mathbf {s}^{\prime \prime T}+\mathbf {s}^{\prime \prime }\mathbf {s}^{T})\right) + \beta ^{2}\sigma _{\tau }^{2} \mathbf {s}^{\prime }\mathbf {s}^{\prime T}\right] \nonumber \\&+ {\sigma _{f}^{2}} \mathbf {I}+ {\sigma _{v}^{2}} \mathbf {I}, \end{aligned}$$

which is similar to the case without fibrosis in (13) but with different proportionality factor and two different random components, corresponding to noise and fibrosis. The eigenvalues will similarly be approximated by:

20 $$\begin{aligned} \lambda _{n} \approx {\left\{ \begin{array}{ll} {\left( \overline{\alpha }^{2}+\sigma _{\alpha }^{2}\right) (E_{s} - \beta ^{2}\sigma _{\tau }^{2} E_{s^{\prime }})+{\sigma _{v}^{2}} + {\sigma _{f}^{2}}} &{} n=1\\ {\left( \overline{\alpha }^{2}+\sigma _{\alpha }^{2}\right) \beta ^{2}\sigma _{\tau }^{2} E_{s'} + {\sigma _{v}^{2}} + {\sigma _{f}^{2}}} &{} n=2\\ {{\sigma _{v}^{2}} + {\sigma _{f}^{2}}} &{} n=3, \cdots , N. \end{array}\right. } \end{aligned}$$

The corresponding EIGDR is approximated by:

21 $$\begin{aligned} \mathcal{R}_{\mathcal{F}} \approx \frac{ E_{s} - \beta ^{2}\sigma _{\tau }^{2} E_{s^{\prime }}}{\beta ^{2}\sigma _{\tau }^{2} E_{s'} + \frac{N({\sigma _{v}^{2}}+ {\sigma _{f}^{2}})}{\left( \overline{\alpha }^{2}+\sigma _{\alpha }^{2}\right) }}. \end{aligned}$$

A summary of eigenvalues and the EIGDR are reported in Table 2 for the four scenarios.

Note that if the inter-signal correlation matrix, ${\mathbf {R}_{u}^{\bullet }=E[\mathbf {u}(n)\mathbf {u}^{T}(n)]}$, with $$\mathbf {u}(n)=\left[ \begin{array}{lll} u_{1}(n)&\cdots&u_{K}(n)\end{array}\right] ^{T}$$, had been computed rather than the intra-signal correlation matrix $\mathbf {R}_{u}$, for the more general case with fibrosis and misalignment, and following a derivation parallel to the one presented in [36], the eigenvalues would have resulted in:

22 $$\begin{aligned} \lambda _{n}^{\bullet } \approx {\left\{ \begin{array}{ll} {\frac{K}{N}\left( \overline{\alpha }^{2}+\sigma _{\alpha }^{2}\right) (E_{s} - \beta ^{2}\sigma _{\tau }^{2} E_{s^{\prime }})+{\sigma _{v}^{2}} + {\sigma _{f}^{2}}} &{} n=1\\ {\frac{K}{N}\left( \overline{\alpha }^{2}+\sigma _{\alpha }^{2}\right) \beta ^{2}\sigma _{\tau }^{2} E_{s'} + {\sigma _{v}^{2}} + {\sigma _{f}^{2}}} &{} n=2\\ {{\sigma _{v}^{2}} + {\sigma _{f}^{2}}} &{} n=3, \cdots , K. \end{array}\right. } \end{aligned}$$

When computing the EIGDRs for this matrix the results are approximately the same as for $\mathbf {R}_{u}$.

In practice, the matrix is estimated as:

23 $$\begin{aligned} \hat{\mathbf {R}}_{u}^{\bullet }= \frac{1}{N}\mathbf {U}^{T}\mathbf {U}, \end{aligned}$$

rather than with the theoretical expectations. From these estimates we observe that matrix $\hat{\mathbf {R}}_{u}^{\bullet }$ is full rank while matrix $\hat{\mathbf {R}}_{u}$ (estimated as in (3)) is not (${N>K}$), circumstance that does not represent a limitation since no matrix inversions are required. In addition, as shown in [33], for the data-estimated autocorrelation matrices with ${N>K}$, ${\lambda _{i}=\frac{N}{K}\lambda _{i}^{\bullet }}$ (${i\le K}$) and ${\lambda _{i}=0}$ (${i> K}$), again showing equivalence of the EIGDR ratios for both matrices when estimated from the available data. Therefore just computational considerations can advise to use one or the other.

**Table 2:** Signal models for non-aligned (NA) and aligned (A) u-EGMs at non-fibrotic (NF) and fibrotic (F) areas, with their respective eigenvalues $\lambda _{k}$ and eigenvalue dominance ratios EIGDR

u-EGM	model	$\lambda _{n}$	EIGDR
NA, NF	$u_{k}(n) = s(n-\tau _{k})+ v_{k}(n)$	$$\lambda _{n} \approx \left\{ \begin{array}{ll} (E_{s} - \sigma _{\tau }^{2} E_{s^{\prime }}) +{\sigma _{v}^{2}}, &{} n=1\\ \sigma _{\tau }^{2} E_{s'}+ {\sigma _{v}^{2}}, &{} n=2 \\ {\sigma _{v}^{2}}, &{} n=3,\cdots ,N \end{array} \right.$$	$\mathcal {R} \approx \frac{E_{s} - \sigma _{\tau }^{2} E_{s^{\prime }}}{\sigma _{\tau }^{2}E_{s'} + N{\sigma _{v}^{2}}}$
A, NF	$u_{k}(n) = s(n)+ v_{k}(n)$	$$\lambda _{n} \approx \left\{ \begin{array}{ll} E_{s} +{\sigma _{v}^{2}}, &{} n=1\\ {\sigma _{v}^{2}}, &{} n=2,\cdots ,N \end{array} \right.$$	${\mathcal{R}^{\mathcal{A}}} \approx \frac{E_{s}}{ N{\sigma _{v}^{2}}}$
NA, F	$u_{k}(n) = \alpha _{k} s(n-\tau _{k}) + f_{k}(n) + v_{k}(n)$	$$\lambda _{n} \approx \left\{ \begin{array}{ll} \left( \overline{\alpha }^{2}+\sigma _{\alpha }^{2}\right) (E_{s} - \beta ^{2}\sigma _{\tau }^{2} E_{s^{\prime }})+{\sigma _{v}^{2}} + {\sigma _{f}^{2}}, &{} n=1\\ \left( \overline{\alpha }^{2}+\sigma _{\alpha }^{2}\right) \beta ^{2}\sigma _{\tau }^{2} E_{s'} + {\sigma _{v}^{2}} + {\sigma _{f}^{2}}, &{} n=2 \\ {\sigma _{v}^{2}} + {\sigma _{f}^{2}}, &{} n=3,\cdots ,N \end{array} \right.$$	${\mathcal{R}_{\mathcal{F}}} \approx \frac{ E_{s} - \beta ^{2}\sigma _{\tau }^{2} E_{s^{\prime }}}{\beta ^{2}\sigma _{\tau }^{2} E_{s'} + \frac{N({\sigma _{v}^{2}}+ {\sigma _{f}^{2}})}{\left( \overline{\alpha }^{2}+\sigma _{\alpha }^{2}\right) }} \nonumber$
A, F	$u_{k}(n) = \alpha _{k} s(n)+ f_{k}(n) + v_{k}(n)$	$$\lambda _{n} \approx \left\{ \begin{array}{ll} \left( \overline{\alpha }^{2}+\sigma _{\alpha }^{2}\right) E_{s} +{\sigma _{v}^{2}} + {\sigma _{f}^{2}}, &{} n=1\\ {\sigma _{v}^{2}} + {\sigma _{f}^{2}}, &{} n=2,\cdots ,N \end{array} \right.$$	${\mathcal{R}_{\mathcal{F}}^{\mathcal{A}}} \approx \frac{ E_{s} }{ \frac{N ({\sigma _{v}^{2}}+ {\sigma _{f}^{2}})}{\left( \overline{\alpha }^{2}+\sigma _{\alpha }^{2}\right) }}$

### EIGDR-based fibrosis markers

According to the previous model, three main differential effects on signal shape, amplitude and arrival times are expected to occur in fibrotic as compared to non-fibrotic tissue:

1. higher morphology dispersion represented by the u-EGM signal component $f_{k}(n)$ and quantified by ${\sigma _{f}^{2}}$;
2. lower and less homogeneous signal amplitudes represented by $\alpha _{k}$ and quantified by $\overline{\alpha }<1$ and $\sigma _{\alpha }^{2}$ (($\overline{\alpha }^{2} + \sigma _{\alpha }^{2}) < 1$);
3. larger inter-signal misalignment within the clique as a result of slowed conduction and represented by delays $\tau _{k}$ with enlarged variance ($\beta ^{2}\sigma _{\tau }^{2}$, $\beta >1$) relative to healthy tissue ($\sigma _{\tau }^{2}$).

In order to determine which ratio to use for discriminating F and NF tissues, we first compare the EIGDR computed with no prior alignment of u-EGMs and no fibrosis, $\mathcal {R}$, with the case with fibrotic tissue, $\mathcal{R}_{\mathcal{F}}$, see Table 2. We observe that $\mathcal{R}_{\mathcal{F}} < \mathcal{R}$, since the numerator in $\mathcal{R}_{\mathcal{F}}$ is smaller than in $\mathcal {R}$ as a result of $\beta$ being larger than one, while the terms in denominator are larger, $\beta ^{2}>1$, ${\sigma _{f}^{2}}>0$ and ($\overline{\alpha }^{2} + \sigma _{\alpha }^{2}) < 1$, as a combination of the three effects introduced by fibrosis. This result suggests the use of the ratio $\mathcal {R}$, which becomes $\mathcal{R}_{\mathcal{F}}$ in fibrosis, with a thresholding strategy to discriminate if the clique is at fibrotic or healthy tissue.

The same analysis can be done when comparing the EIGDR with prior alignment of u-EGMs and fibrosis, $\mathcal{R}_{\mathcal{F}}^{\mathcal{A}}$, with respect to its counterpart with no fibrosis, $\mathcal{R}^{\mathcal{A}}$. In this case, only the terms ${\sigma _{f}^{2}}>0$ and ($\overline{\alpha }^{2} + \sigma _{\alpha }^{2}) < 1$ are responsible of the difference, since $\sigma _{\tau }^{2}$ has already been compensated for with alignment, resulting in $\mathcal{R}_{\mathcal{F}}^{\mathcal{A}} < \mathcal{R}^{\mathcal{A}}$. This suggests that $\mathcal{R}^{\mathcal{A}}$ may also be used as a thresholding strategy to discriminate fibrotic from healthy tissue.

In order to study which of the two options, $\mathcal {R}$ or $\mathcal{R}^{\mathcal{A}}$, is more sensitive to fibrotic tissue characteristics, we analyze how the difference between $\mathcal {R}$ and $\mathcal{R}^{\mathcal{F}}$ evolves by varying $\sigma _{\tau }^{2}$. For that purpose, we compute the ratio $\Delta \mathcal{R}_{\mathcal{F}}$, which under the proposed signal model can be approximated by:

24 $$\begin{aligned} \Delta \mathcal{R}_{\mathcal{F}} = \frac{\mathcal {R}}{\mathcal{R}_{\mathcal{F}}}\approx \frac{\beta ^{2}\sigma _{\tau }^{2} E_{s'} + \frac{N({\sigma _{v}^{2}}+ {\sigma _{f}^{2}})}{\left( \overline{\alpha }^{2}+\sigma _{\alpha }^{2}\right) }}{ \sigma _{\tau }^{2} E_{s'} + N{\sigma _{v}^{2}}}. \end{aligned}$$

Its partial derivative with respect to $\sigma _{\tau }^{2}$ is:

25 $$\begin{aligned} \frac{\partial \Delta \mathcal{R}_{\mathcal{F}}}{\partial \sigma _{\tau }^{2}} \approx \frac{ - E_{s'} N \left( {\sigma _{f}^{2}} + {\sigma _{v}^{2}} \left( 1-\beta ^{2}\left( \overline{\alpha }^{2}+\sigma _{\alpha }^{2}\right) \right) \right) }{\left( \overline{\alpha }^{2}+\sigma _{\alpha }^{2}\right) \left( \sigma _{\tau }^{2}E_{s'} + N{\sigma _{v}^{2}}\right) ^{2}}. \end{aligned}$$

Typically $\left( 1-\beta ^{2}\left( \overline{\alpha }^{2}+\sigma _{\alpha }^{2}\right) \right)$ $>0$ in fibrosis since conduction velocity reduction is less prominent ($\beta \approx 2$ for high fibrosis, [38, 39]) than voltage attenuation ($\overline{\alpha }\approx 0.3$ [12]) and consequently the product $\beta ^{2}\overline{\alpha }^{2}<1$. This results in $\frac{\partial \Delta \mathcal{R}_{\mathcal{F}}}{\partial \sigma _{\tau }^{2}} < 0$, meaning that the lower the misalignment $\sigma _{\tau }^{2}$ the larger $\Delta \mathcal{R}_{\mathcal{F}}$, implying higher EIGDR differences between fibrotic, $\mathcal{R}_{\mathcal{F}}$, and healthy, $\mathcal {R}$, tissue. This justifies the advantage of pre-aligning u-EGMs in the cliques before EIGDR calculations, since the higher the misalignment $\sigma _{\tau }^{2}$, the lower $\Delta \mathcal{R}_{\mathcal{F}}$ and consequently the capacity of $\mathcal {R}$ to discriminate between fibrosis and non-fibrosis, and suggests that $\mathcal{R}^{\mathcal{A}}$ is better suited fibrosis marker than $\mathcal {R}$.

Alternatively, we considered the ratio $\Delta \mathcal{R}^{\mathcal{A}}$ of EIGDR computed from perfectly aligned u-EGMs with respect to misaligned original u-EGM signals, representing the gain in eigenvalue concentration produced by alignment:

26 $$\begin{aligned} \Delta {\mathcal{R}^{\mathcal{A}}} = \frac{\mathcal{R}_{\mathcal{F}}^{\mathcal{A}}}{\mathcal{R}_{\mathcal{F}}}\approx \frac{E_{s} \left( \beta ^{2}\sigma _{\tau }^{2} \left( \overline{\alpha }^{2}+\sigma _{\alpha }^{2}\right) E_{s'} + N({\sigma _{v}^{2}}+ {\sigma _{f}^{2}}) \right) }{ \left( N({\sigma _{v}^{2}}+ {\sigma _{f}^{2}})\right) \left( E_{s} - \beta ^{2}\sigma _{\tau }^{2} E_{s^{\prime }}\right) }. \end{aligned}$$

This expression has been estimated for the more general case including fibrosis (${\sigma _{f}^{2}} > 0$), so expressions ${\mathcal{R}^{\mathcal{A}}_{\mathcal{F}}}$ and ${\mathcal{R}_{\mathcal{F}}}$ are used. Nevertheless, it can certainly be computed at cliques on any tissue, fibrotic or non-fibrotic, and its theoretical value when no fibrosis is present can be retrieved from (26) just by making $\sigma _{f}=0$, $\beta \mathbf =1$, $\overline{\alpha }=1$, and $\sigma _{\alpha }=0$. Sensitivity of $\Delta \mathcal{R}^{\mathcal{A}}$ to fibrosis has been analyzed by deriving (26) with respect to the fibrosis-induced parameters. Therefore, deriving with respect to ${\sigma _{f}^{2}}$ to see how $\Delta \mathcal{R}^{\mathcal{A}}$ depends on the level of fibrosis, we obtain:

27 $$\begin{aligned} \frac{\partial \Delta \mathcal{R}^{\mathcal{A}}}{\partial {\sigma _{f}^{2}}} \approx \frac{ - E_{s} N \beta ^{2}\sigma _{\tau }^{2} \left( \overline{\alpha }^{2}+\sigma _{\alpha }^{2}\right) E_{s'} }{ \left( N({\sigma _{v}^{2}}+ {\sigma _{f}^{2}})\right) ^{2} \left( E_{s} - \beta ^{2}\sigma _{\tau }^{2} E_{s^{\prime }}\right) }. \end{aligned}$$

For small delays $\tau _{k}$, ${E_{s} \gg \beta ^{2}\sigma _{\tau }^{2} E_{s^{\prime }}}$, this expression results in ${\frac{\partial \Delta \mathcal{R}^{\mathcal{A}}}{\partial {\sigma _{f}^{2}}} < 0}$, implying that $\Delta \mathcal{R}^{\mathcal{A}}$ gets reduced if the fibrotic component ${\sigma _{f}^{2}}$ increases and suggesting the possibility of using $\Delta \mathcal{R}^{\mathcal{A}}$ as a fibrosis marker, like ${\mathcal {R}}$ and ${\mathcal{R}^{\mathcal{A}}}$. This behavior is also corroborated by computing the derivative of $\Delta \mathcal{R}^{\mathcal{A}}$ with respect to $\overline{\alpha }^{2}$, taking into account u-EGM amplitude reduction in fibrosis:

28 $$\begin{aligned} \frac{\partial \Delta \mathcal{R}^{\mathcal{A}}}{\partial \overline{\alpha }^{2}} = \frac{ E_{s} \beta ^{2}\sigma _{\tau }^{2} E_{s'}}{ \left( N({\sigma _{v}^{2}}+ {\sigma _{f}^{2}})\right) \left( E_{s} - \beta ^{2}\sigma _{\tau }^{2} E_{s^{\prime }}\right) }. \end{aligned}$$

This expression results ${>0}$ for small delays $\tau _{k}$, confirming that the larger the fibrosis (i.e., the smaller $\overline{\alpha }^{2}$), the smaller $\Delta \mathcal{R}^{\mathcal{A}}$.

Nevertheless, under the same assumptions, the derivative of $\Delta \mathcal{R}^{\mathcal{A}}$ with respect to $\beta ^{2}$ results in $\frac{\partial \Delta \mathcal{R}^{\mathcal{A}}}{\partial \beta ^{2}} > 0$:

29 $$\begin{aligned} \frac{\partial \Delta \mathcal{R}^{\mathcal{A}}}{\partial {\beta }^{2}} \approx \frac{ E_{s} \sigma _{\tau }^{2} E_{s'}}{ \left( E_{s} - \beta ^{2}\sigma _{\tau }^{2} E_{s^{\prime }}\right) ^{2}} \left( 1+ \frac{E_{s}(\overline{\alpha }^{2}+\sigma _{\alpha }^{2})}{N ({\sigma _{v}^{2}}+{\sigma _{f}^{2}})}\right) , \end{aligned}$$

meaning that the larger the reduction of velocity due to fibrosis (i.e., the higher $\beta$), the greater $\Delta \mathcal{R}^{\mathcal{A}}$, thus showing an opposite effect.

However, as already said before, we expect that fibrosis effects on u-EGM amplitude and morphology, expressed by $\overline{\alpha }^{2}$ and ${\sigma _{f}^{2}}$, respectively, are much more marked than those on conduction velocity given by $\beta ^{2}$ [40]. Therefore, we expect the index $\Delta \mathcal{R}^{\mathcal{A}}$ to be reduced when fibrosis is more severe.

According to this analysis, three different EIGDR-based metrics revealed to be sensitive to the presence of fibrosis and therefore suitable to distinguish between fibrotic and non-fibrotic areas: $\mathcal {R}$, $\mathcal{R}^{\mathcal{A}}$, which can be interpreted as measurements of the shape homogeneity of the u-EGMs before and after time alignment, respectively, and the ratio between both, $\Delta \mathcal{R}^{\mathcal{A}}$. Maps of $\mathcal {R}$, $\mathcal{R}^{\mathcal{A}}$ and $\Delta \mathcal{R}^{\mathcal{A}}$ were computed by processing the complete MEA in the two clique sizes considered in the simulation study. Each map consists of color-coded pixels, representing EIGDR value at each clique. The $2\times 2$ configuration provides one EIGDR value for each square group of four electrodes with diagonal vertices at (*i*, *j*), and $(i+1,j+1),i,j \in \{1,\cdots ,14\}$, giving a total of $14\times 14$ pixel maps for each proposed marker. The $3\times 3$ clique provides one EIGDR value at each squared group of nine electrodes with diagonal vertices at (*i*, *j*) and $(i+2,j+2)$, $i,j\in \{1,\cdots ,13\}$, resulting in maps of $13\times 13$ pixels, for $\mathcal {R}$, $\mathcal{R}^{\mathcal{A}}$ and $\Delta \mathcal{R}^{\mathcal{A}}$.

### EIGDR with variable electrode-to-tissue distance

When we introduce variable electrode-to-tissue distance, we need to modify the model by replacing *s*(*n*) with $\mu _{k} s(n)$, being $\mu _{k}$ a random variable with mean $\overline{\mu }=1$ and variance $\sigma _{\mu }^{2}$ indexing all the $\mu _{i,j}$ within the clique. Similar analysis as in previous subsections leads to obtain:

30 $$\begin{aligned} \mathcal{R}^{\mathcal{A}} \approx \frac{E_{s}(1+\sigma _{\mu }^{2})}{N{\sigma _{v}^{2}}} \ \ \quad ,\quad \mathcal{R}^{\mathcal{A}}_{\mathcal{F}} \approx \frac{ E_{s}(1+\sigma _{\mu }^{2})}{\frac{N({\sigma _{v}^{2}}+ {\sigma _{f}^{2}})}{\left( \overline{\alpha }^{2}+\sigma _{\alpha }^{2}\right) }}, \end{aligned}$$

which just introduces a multiplying factor, $(1+\sigma _{\mu }^{2})$, with respect to the ratios in (11) and (18), equal in both ratios, so preserving the fibrosis stratification value of $\mathcal{R}^{\mathcal{A}}$ biomarker. This occurs in contrast to b-EGM peak-to-peak marker, $V_{i,j}^{b}$, which is largely modified by the variable electrode-to-tissue distance, but a random way at each electrode, reducing its value as a stratification marker. Analogously:

31 $$\begin{aligned} \mathcal {R} \approx \frac{(E_{s} - \sigma _{\tau }^{2} E_{s^{\prime }})(1+\sigma _{\mu }^{2})}{\sigma _{\tau }^{2} E_{s^{\prime }}(1+\sigma _{\mu }^{2}) + N{\sigma _{v}^{2}}} , \end{aligned}$$

32 $$\begin{aligned} \mathcal{R}_{\mathcal{F}} \approx \frac{ (E_{s} - \beta ^{2}\sigma _{\tau }^{2} E_{s^{\prime }})(1+\sigma _{\mu }^{2})}{\beta ^{2}\sigma _{\tau }^{2} E_{s'} (1+\sigma _{\mu }^{2})+ \frac{N({\sigma _{v}^{2}}+ {\sigma _{f}^{2}})}{\left( \overline{\alpha }^{2}+\sigma _{\alpha }^{2}\right) }}, \end{aligned}$$

which approximately result in the same multiplying factor $(1+\sigma _{\mu }^{2})$ with respect to ratios (15) and (21). Note that for small $\sigma _{\tau }$, ${N{\sigma _{v}^{2}}>> \sigma _{\tau }^{2} E_{s^{\prime }}}$ and then the approximation of a multiplying factor relating fixed with variable electrode-to-tissue distance holds. This again shows that $\mathcal {R}$ preserves the fibrotic stratification value in variable electrode-to-tissue distance situations. The same analysis also applies to the ratio $\Delta {\mathcal{R}^{\mathcal{A}}}$.

Also note that in presence of more complex fibrillatory propagation patterns, changes occurring in the u-EGM morphology from electrode to electrode can initially be thought as a planar wave propagating in different directions. This will also result in an extra *k*-dependent amplitude component into the $s_{k}(n-\tau _{k})$ signal in the model of (8), depending of the angle of the planar wave, and thus also evidence not to largely affect the EIGDR.

### EIGDR in real data from PentaRay^®^

Values of $\mathcal {R}$, $\mathcal{R}^{\mathcal{A}}$ and $\Delta \mathcal{R}^{\mathcal{A}}$ were also computed within the four- and five-electrode cliques considered at each mapping point acquired by the PentaRay^®^ catheter. In order to quantify the atrial activity related dispersion, an atrial depolarization window of 100 ms fixed length (${N= 100}$) was extracted from the last recorded activation at each recording site. Therefore, the proposed EIGDR-based markers were calculated using windowed signals in each clique, aligning them when required as explained in Section 2.5.

### Voltage-based fibrosis markers

We also considered bipolar voltage maps based on the peak-to-peak amplitudes ${V_{i,j}^{b\text{- }x}}$ and ${V_{i,j}^{b\text{- }y}}$ of the b-EGMs in each of the two MEA directions, $b_{i,j}^{x}(n)$, ${i\in \{1,\cdots ,14\}}$, ${j\in \{1,\cdots ,15\}}$, and ${b_{i,j}^{y}(n)}$, ${i\in \{1,\cdots ,15\}}$, ${j\in \{1,\cdots ,14\}}$, as well as on their maximum ${V_{i,j}^{b\text{- }m}=\max \{V_{i,j}^{b\text{- }x}, V_{i,j}^{b\text{- }y}\}}$, ${i\in \{1,\cdots ,14\}}$, ${j\in \{1,\cdots ,14\}}$. These peak-to-peak amplitude-based maps were considered and their performance for fibrosis detection were compared against EIGDR maps. Each color-coded pixel bipolar map presents the same resolution as ${2\times 2}$ cliques EIGDR maps, providing ${14\times 14}$ pixels when processing the whole MEA.

Regarding clinical data, for each mapping point we derived b-EGMs along the PentaRay^®^ catheter branches from filtered u-EGMs. Peak-to-peak amplitudes were computed using atrial depolarization windows extracted from the last recorded activation of b-EGMs at each bipole.

### Performance assessment for fibrosis detection

Both EIGDR and bipolar mapping strategies were estimated and tested for each noisy realization $u^{q}_{i,j}(n)$, ${i,j\in \{1,\cdots ,15 \}}$, $q\in \{1,\cdots ,2000\}$ considered in this study. For each map type, results are reported by aggregating the three different MEA orientations. This aggregated version represents a scenario where the relative angle of the propagation direction with respect to the catheter was not known a priori, thus being more realistic.

**Fig. 7:**
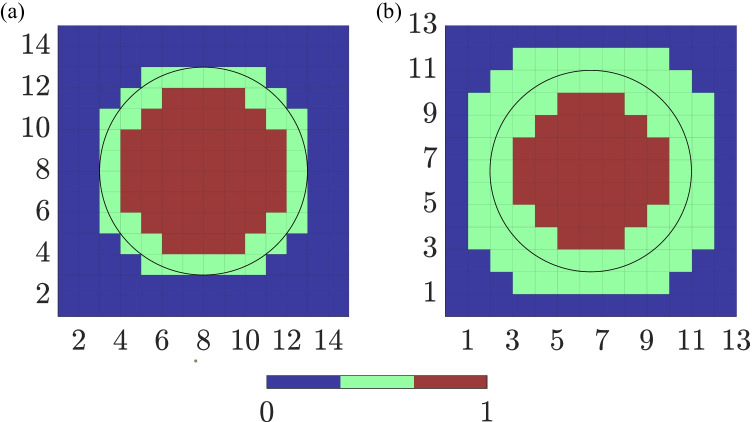
(a) $14 \times 14$ and (b) $13 \times 13$ ground-truth masks for evaluating fibrosis detection ability of maps performed with 2$\times$2 and 3$\times$3 cliques, respectively. Green squares represent the pixels corresponding to cliques with some electrodes inside and some outside the fibrotic patch, i.e. those cliques lying in the border separating the fibrotic patch from non-fibrotic tissue, which were excluded from the evaluation

In order to quantitatively evaluate the ability of the different maps as markers for fibrosis detection, i.e. in discriminating pixels associated to the fibrotic patch from those related to non-fibrotic tissue, receiver operating characteristic (ROC) curves were used. Two ground-truth masks were created for that purpose, with the resolution of $14 \times 14$ and $13 \times 13$ maps, by labeling whether a clique (or an electrode pair in case of bipolar maps) lies within the fibrotic or the non-fibrotic area. In a first analysis, the ${14 \times 14}$ ground-truth mask was created by assigning value 1 if the four electrodes within a $2\times 2$ clique lie in the fibrotic area, and value 0 if the four electrodes lie in the non-fibrotic area. In a similar way, the ${13\times 13}$ ground-truth mask was created by considering if the nine electrodes within a $3\times 3$ clique fully lie or not in fibrotic/non-fibrotic tissue. Cliques with some electrodes inside and some outside the fibrotic patch were not labeled and therefore discarded in the evaluation. The two ground-truth masks used in this study are shown in Fig. 7(a) and (b), for evaluating 14$\times$14 maps (both EIGDR and bipolar) and 13$\times$13 maps, respectively. Then, in a further analysis, two binary ${14 \times 14}$ and $13 \times 13$ ground-truth masks including those mixed cliques with electrodes inside and outside fibrosis region were considered for the evaluation. For that purpose, cliques whose distance between their central point and the center of the fibrotic patch was shorter than the radius of the patch were labeled as fibrotic. On the contrary, when this distance was longer than the radius, corresponding cliques were classified as non-fibrotic. For each EIGDR and bipolar mapping strategy, ROC curves were computed by varying the threshold for fibrosis identification, obtaining sensitivity and specificity in the detection of the fibrotic area [41]. In this context, *true positive* denotes the number of cliques correctly identified as fibrotic, *false negative* represents the number of missed cliques in the fibrotic area, *true negative* stands for the number of cliques correctly identified as non-fibrotic and *false positive* is the number of cliques incorrectly detected as fibrotic. The maximum detection accuracy (*ACC*), defined as the highest number of correctly identified cliques (fibrosis or non-fibrosis) divided by the total number of assessed cliques, was used as a measure of the overall fibrosis detection ability of each map. Values of *ACC*, as well as of the threshold corresponding to *ACC*, were computed for each map aggregated version considered in this work. Averaged results over the noisy realizations were then computed and evaluated for performance measurements.

In the clinical data analysis, at each F and NF mapping point, median values of the three ratios $\mathcal {R}$, $\mathcal{R}^{\mathcal{A}}$ and $\Delta \mathcal{R}^{\mathcal{A}}$ were calculated over the six cliques considered. Analogously, the median and maximum values, $V^{b}$ and $V^{b\text {-}m}$, respectively, among the five peak-to-peak bipolar amplitudes computed along the catheter branches and associated with the bipoles closest to the its center ((3,4), (7,8), (11,12), (15,16) and (19,20), according to Fig. 4), were computed at each mapping point in fibrosis and healthy tissue. In order to compare markers between F and NF tissues, median and interquartile range (IQR) over all the EIGDR-based indices and bipolar amplitudes were computed at both F and NF points, separately, as well as the *p*-values of the right-tailed Wilcoxon rank-sum test referring to the comparison of the metrics between the two areas. Finally, median and IQR of EIGDR indices and bipolar amplitudes were calculated over the six cliques and the five innermost bipoles, respectively, of all mapping points considered, at both F and NF areas, as well as their *p*-values referring to the global comparison of the metrics between those F and NF areas.

## Results

### Analysis of simulated data

**Fig. 8:**
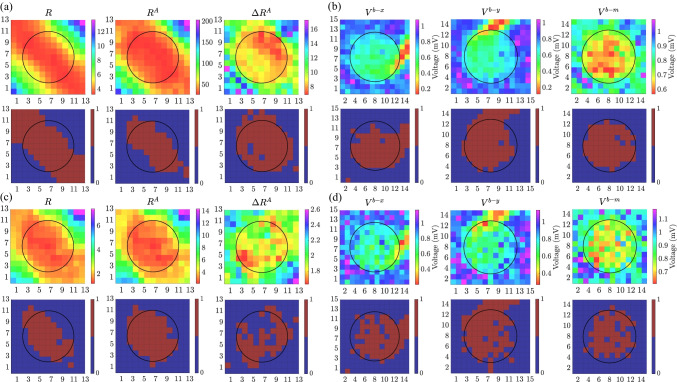
Upper panels: maps of $\mathcal {R}$, $\mathcal{R}^{\mathcal{A}}$, $\Delta \mathcal{R}^{\mathcal{A}}$ from 3$\times$3 cliques and bipolar voltage maps $V^{b\text {-}x}$, $V^{b\text {-}y}$, $V^{b\text {-}m}$, for $\Psi$ = $45^{\circ }$, performed assuming a variable electrode-to-tissue distance and noise free ((a) and (b)) and noisy ((c) and (d), with noise level $\sigma _{v}$ = 46.4 $\mu V$) u-EGMs. Lower panels: detected fibrotic areas (brown), using the thresholds that maximize detection accuracy of each map

An example of the mapping strategies (computed with 3$\times$3 cliques) for $\Psi = 45^{\circ }$, variable electrode-to-tissue distance and without noise, is shown for EIGDR and bipolar maps at upper panels in Fig. 8(a) and (b), respectively. When noise is added at level of $\sigma _{v} = 46.4 \ \mu V$, results for one of the two thousand noisy realizations are presented at Fig. 8(c) and (d). In the lower panels, the fibrotic areas identified by using the thresholds that maximize the *ACC* are shown for each mapping strategy. Blue (brown) color inside the circle encompassing fibrotic patch denotes false negative (true positive), while outside denotes true negative (false positive) detection, respectively.

We reported values of $\mathcal {R}$, $\mathcal{R}^{\mathcal{A}}$ and $\Delta \mathcal{R}^{\mathcal{A}}$ computed from noisy (${\sigma _{v}=46.4 \ \mu V}$) u-EGMs in the non-fibrotic clique ${(i,j) = (3,3)}$: ${\mathcal {R} = 3.39}$, ${\mathcal{R}^{\mathcal{A}} = 7.76}$ and ${\Delta \mathcal{R}^{\mathcal{A}} = 2.29}$, as well as in the fibrotic clique ${(i,j) = (8,7)}$: ${\mathcal {R} = 1.36}$, ${\mathcal{R}^{\mathcal{A}} = 2.44}$ and ${\Delta \mathcal{R}^{\mathcal{A}} = 1.79}$, which are consistent with derivations of the models presented in Section 2.7.

Results in this example illustrate that EIGDR maps performed from noise-free time-aligned u-EGMs, $\mathcal{R}^{\mathcal{A}}$ and $\Delta \mathcal{R}^{\mathcal{A}}$, plotted at central and rightmost columns in Fig. 8(a), respectively, present fibrosis detection performance comparable to bipolar maps obtained as the maximum voltage of both MEA directions, $V^{b\text {-}m}$, shown at rightmost column in Fig. 8(b). However, when u-EGMs are affected by noise, EIGDR maps (upper row at Fig. 8(c)) are more robust than bipolar maps (upper row at Fig. 8(d)), being $\mathcal{R}^{\mathcal{A}}$ the one showing the best fibrosis discrimination performance.

*ACC* values of all mapping strategies considered in this study are summarized in Table 3, assuming fixed or variable distance between MEA and tissue, and five different noise levels (reported as standard deviations $\sigma _{v}$ and average peak-to-peak amplitudes $\overline{V}_{pp,v}$). For each map, thresholds having these maximum detection accuracy values were also reported in Table 4, where they were given in voltage units in case of b-EGM amplitude-based maps. Both *ACC* and threshold values were calculated and reported by aggregating the three catheter orientations with respect to the propagation direction. Despite this, bipolar voltage maps exhibit performance strongly dependent on the relative orientation between MEA and propagation direction (e.g., ${ACC = 68.7 \%}$ and ${ACC = 90.8 \%}$ for $V^{b\text {-}x}$ and $V^{b\text {-}y}$, respectively, for fixed catheter-to-tissue distance and ${\sigma _{v} = 0.0 \ \mu V}$). For ${\sigma _{v} = 46.4 \ \mu V}$, $V^{b\text {-}y}$ and $V^{b\text {-}m}$ are more affected by noise than EIGDR maps, both for fixed and variable electrode-to-tissue distance.

The selection of the thresholds for the EIGDR implies another challenge. This can be addressed by observing that values reached by $\mathcal{R}^{\mathcal{A}}$ at healthy tissue (11) can be obtained as the ratio between the estimated energy $\hat{E}_{s}$ and *N* times the estimate of the noise variance $\hat{\sigma }_{v}^{2}$, ${\hat{\mathcal {R}}^{\mathcal {A}}=\hat{E}_{s}/(N \hat{\sigma }_{v}^{2})}$. The value of $\hat{E}_{s}$ can be estimated from the data (e.g., by averaging the energy of the u-EGMs in the clique), while the noise variance $\hat{\sigma }_{v}^{2}$ can be estimated as the u-EGM signal variance in areas electrically silent. The threshold, $\mathcal {T}$, can be fixed to a value ${\mathcal {T}=\hat{E}_{s}/(N \hat{\sigma }_{v}^{2})-\Delta }$, where $\Delta$ will control the trade-off between required sensitivity and specificity: small $\Delta$ will provide high sensitivity and low specificity, and the reverse for large $\Delta$. Note that from results reported in this subsection for a non-fibrotic site such as $(i,j)=(3,3)$ with noise ${\sigma _{v}=46.4 \mu V}$, we measured ${\hat{\mathcal {R}}^{\mathcal {A}}=7.76}$, while the optimum threshold reported in Table 4 for this noise level is 3.5, corresponding to a $\hat{\Delta }=4.26$, which can be taken as a reference value. Similar analysis for the same noise level gives $\Delta$ values estimates of 1.7 for ${\mathcal {R}}$ and 0.39 for ${\Delta \mathcal {R}^{\mathcal {A}}}$, as quantities to subtract to the estimates of ${\hat{\mathcal {R}}}$ and ${\hat{\Delta }\mathcal {R}^{\mathcal {A}}}$ at non-fibrotic areas to derive usable threshold values in real clinical settings. These estimates will require additionally an estimate of the $\hat{\sigma }_{\tau }$ and $\hat{E}_{s^{\prime }}$, see Eqs. (15) and (26), which can be computed, e.g., from the standard deviation of estimated delays in a clique and from the derivative of the aligned and averaged u-EGMs in the clique, respectively.

Bipolar voltage map $V^{b\text {-}m}$ identifies the fibrotic area with an *ACC* of 96.2% when distance between MEA and tissue is fixed and u-EGMs are not affected by noise (${\sigma _{v} = 0.0 \ \mu V}$). Nevertheless, this performance reduces to ${ACC = 92.5\pm 1.1 \%}$ when the electrode-to-tissue distance is variable, and further when increasing noise level, reaching values 86.9±1.1% and 86.1±1.2% for the highest noise level (${\sigma _{v} = 46.4 \ \mu V}$), in case of fixed and variable electrode-to-tissue distance, respectively. On the other hand, $\mathcal{R}^{\mathcal{A}}$ performed with 3$\times$3 cliques is more robust to the effect of variable distance than $V^{b\text {-}m}$, presenting ${ACC = 92.1 \%}$ and ${ACC = 92.3\pm 0.8 \%}$ for fixed and variable catheter-to-tissue distance, respectively. In addition, it achieves ${ACC = 94.2\pm 1.6 \%}$ when ${\sigma _{v} = 46.4 \ \mu V}$, for both fixed and variable distance scenarios, being consistent with example in Fig. 8. The same behavior has been observed when studying the three MEA orientations separately. For the highest noise level under test, and with 3$\times$3 cliques, $\mathcal{R}^{\mathcal{A}}$ achieves greater *ACC* values than $V^{b\text {-}m}$. In particular, $\mathcal{R}^{\mathcal{A}}$ reaches 95±2%, 95±3%, and 95±3% for $\Psi =0^{\circ }$, $30^{\circ }$, and $45^{\circ }$, respectively, both with fixed and variable electrode-to-tissue distances, while $V^{b\text {-}m}$ reaches 88±2%, 89±2%, and 90±2% with fixed distance, and 87±2%, 88±2% and 89 ±2%, with variable distance, for $\Psi = 0^{\circ }$, $30^{\circ }$, and $45^{\circ }$, respectively. If the evaluation is performed without exclusion of cliques which have electrodes inside and outside the fibrotic patch, $\mathcal{R}^{\mathcal{A}}$ still provides higher *ACC* (83.0 ± 1.5%) than $V^{b\text {-}m}$ (81.2 ± 1.21%), in the largest noise contamination and with variable electrode-to-tissue distance. Moreover, when u-EGMs are not affected by noise, *ACC* goes from 80.2 % to 80.1 ± 0.6 % for $\mathcal{R}^{\mathcal{A}}$ while *ACC* reduces from 90.8 % to 87.2 ± 1.0 % for $V^{b\text {-}m}$, from fixed to variable electrode-to-tissue distance.

### Analysis of clinical data

Table 5 shows the median values and IQR of the different biomarkers computed at nineteen mapping points at fibrotic (F) and other nineteen at non-fibrotic (NF) areas. Two of them are depicted in Fig. 6. It can be observed that median values related to $\mathcal {R}$ and $\mathcal{R}^{\mathcal{A}}$ indices evaluated at NF tissue are greater than their counterparts at F points. When u-EGMs are not previously time aligned, $\mathcal {R}$ shows the following median [IQR] values: 2.45 [0.80] vs. 2.22 [1.47], at NF vs. F points, respectively. The same occurs when considering $\mathcal{R}^{\mathcal{A}}$ (7.35 [5.62] vs. 6.18 [4.08]), which revealed to be significantly lower at F than at NF areas (Wilcoxon rank-sum test, *p*-value<0.05).

On the other hand, when considering each clique or bipolar measurement independently, as reported in Table 6, EIGDR markers based on the alignment of u-EGMs showed to be significantly greater at NF than their counterparts at F areas, assuming the following median [IQR] values: 7.42 [6.74] and 2.67 [3.25] vs. 5.85 [5.62] and 2.17 [3.12] for $\mathcal{R}^{\mathcal{A}}$ and $\Delta \mathcal{R}^{\mathcal{A}}$ at non-fibrotic and fibrotic tissue, respectively. These results are consistent with the theoretical model and overtake fibrosis discrimination performance of $V^{b}$ (Wilcoxon rank-sum test, *p*-value<0.05).

## Discussion

### Clinical significance of the work

Detection of atrial fibrosis is capital for guiding catheter ablation strategies in AF. The typical intra-procedural assessment of atrial fibrosis by means bipolar voltage thresholding presents well-established limitations related to catheter-wavefront orientation, catheter-tissue contact, electrodes size and inter-electrode spacing, thus limiting its reliability as surrogate of fibrosis. Besides this, it is well-known that when using threshold-based approach, EGM morphology information and time relationship among adjacent electrodes are missing. Despite late gadolinium enhancement-magnetic resonance imaging represents the only non-invasive tool for atrial fibrosis diagnosis, its reproducibility remains under debate [42], as well as its utility in clinical settings [43].

In this work, we proposed u-EGMs eigenvalue dominance ratios (EIGDR) to quantify voltage waveform dispersion and investigated their performance as markers in discriminating fibrotic and non-fibrotic areas, by using a 2D simulated tissue including diffuse fibrosis. The hypothesis behind this approach is that underlying fibrosis in the atrium is reflected not only in the reduction of the waveform amplitude but also in the increased inter-signal dispersion in cliques of nearby electrodes, and that this dispersion will be insensitive to electrode-to-tissue distance as opposite to b-EGM amplitudes.

### Performance evaluation of fibrosis markers with simulated data

We analyzed maps computed from noise-free u-EGMs, as well as from u-EGMs corrupted by homogeneous noise levels. As a first step, the distance between each electrode of a square MEA and tissue was assumed to be fixed at 1 mm. Then, in a further analysis, that distance was assumed to be variable following a normal distribution, so as to better approach the real situation where there is no guarantee of maintaining a perfect contact during the mapping.

Our results show that reducing misalignment among u-EGMs within the clique improves fibrosis detection ability of the proposed EIGDR-based index. This is in agreement with other studies where time alignment of b-EGMs has shown to be beneficial for electroanatomical mapping strategies robustness [8]. The index $\mathcal{R}^{\mathcal{A}}$ provides comparable fibrosis detection accuracy to the one of maximum bipolar voltage maps when u-EGMs are not affected by noise, and better when high noise levels are present (${\sigma _{v} \ge 23.2 \ \mu V}$), for both fixed and variable electrode-to-tissue distances.

Results obtained by considering the three MEA orientations separately reinforce the consideration of $\mathcal{R}^{\mathcal{A}}$ as an index worth to be analyzed in extended studies with real recordings for discriminating between fibrotic and normal areas, also pointing out the larger impact of catheter orientation in bipolar amplitudes than in EIGDR metrics.

In addition, if the evaluation is performed without exclusion of cliques which have electrodes inside and outside the fibrotic patch, similar conclusions, with reduced difference ranges, can be drawn. The ratio $\mathcal{R}^{\mathcal{A}}$ still shows higher *ACC* in the largest noise contamination and with variable electrode-to-tissue distance. Moreover, EIGDR-based markers reveal to be more robust to the effect of variable distance than bipolar maps, especially when u-EGMs are not affected by noise.

Regarding threshold values corresponding to the maximum fibrosis detection accuracy, our findings reveal that the thresholds needed to maximize accuracy of bipolar maps $V^{b\text {-}y}$ and $V^{b\text {-}m}$ are greater than the one typically used in clinical settings (0.5 $\mu V$), whereas $V^{b\text {-}x}$ presents lower voltage threshold. This is explained by the fact that there is no projection of wavefront propagation along the *x*-axis of the MEA when propagation orientation is $\Psi = 0^{\circ }$.

### Performance evaluation of fibrosis markers with clinical data

In the present study, we also tested the ability of the EIGDR-based markers to characterize the fibrotic substrate considering different mapping points acquired with a PentaRay^®^ catheter within fibrotic and non-fibrotic areas over the LA anatomical map, using MRI as reference for that purpose.

The electrode clique organization, referred to a fixed structure catheter like the Advisor^TM^ HD Grid, was extended to a flexible structure catheter like the PentaRay^®^, where the inter-electrode spacing within the clique may vary at different acquisition points. Nevertheless, this does not represent a problem for the proposed method, as it is not dependent on electrode orientation.

Preliminary findings obtained from real u-EGMs in this paper reveal that the ratio based on time-aligned u-EGMs $\mathcal{R}^{\mathcal{A}}$ is the only EIGDR-based marker between F and NF mapping points, also showing better discrimination power than bipolar amplitudes $V^{b}$ and $V^{b\text {-}m}$ typically used in clinical settings. In addition, $\mathcal{R}^{\mathcal{A}}$, together with $\Delta \mathcal{R}^{\mathcal{A}}$, proved to be the only indices capable to globally discriminate fibrosis from non-fibrotic tissue, regardless of the mapping points and cliques/bipoles considered at each of them.

## Limitations

Several limitations of this study need to be highlighted. First, we simulated a single scenario reproducing a simple propagation pattern in a 2D atrial model, which largely simplifies the real 3D anatomical and electrophysiological situations.

Although a single plane wavefront that propagates in a homogeneous tissue lends itself well to approximating the propagation during pacing, the previous considerations do not allow us to extend quantitatively the results to other propagation patterns, such as circular waves, wave collisions, reentrant wave fronts, among others, and model conditions, including conduction anisotropy or patchy fibrosis. Nevertheless, even if it is well-established that the underlying propagation pattern strongly influences EGMs morphology and their spatiotemporal information, we expect that it does not largely affect the local EIGDR computation. This is because we hypothesize that the correlation between the presence of fibrosis and the morphology dispersion of signals in electrode cliques is well modeled by a waveform assumed to be locally plane and homogeneous, irrespective of the global waveform distribution across the complete tissue. For the same reasons, we considered that the EIGDR approach would not be largely affected by the shape and size of the fibrotic patch. Note that the proposed intra-clique time alignment of the u-EGMs compensates the effect of different u-EGM arrival times on EIGDR. This leaves EIGDR to mostly represent spatial relationships differences among u-EGMs within each clique.

In this work, only the effect of broad-band noise affecting u-EGMs was considered, while specific periodic types of noise were not considered. It must be noted that far-field disturbances due to ventricular depolarization did not occur during atrial activation in sinus rhythm.

Lastly, results presented with real signals represent a proof of concept, but increased sample size need to be considered in order to elucidate whether the use of the EIGDR-based approach is advantageous for fibrosis detection in clinical settings.

**Table 3:** *ACC* of EIGDR and bipolar amplitude maps, reported jointly for the three MEA orientations and different scenarios, with fixed (FD) or variable (VD) electrode-to-tissue distance, corrupting u-EGMs with noise levels ${\sigma _{v}\in \{0.0, 5.8, 11.6, 23.2, 46.4\} \ \mu V}$ (${\overline{V}_{pp,v}\in \{0.0, 24.2, 48.4, 96.7, 193.5\} \ \mu V}$). *ACC* values are presented as mean ± standard deviation except for fixed electrode-to-noise distance and $\sigma _{v}$ ($\overline{V}_{pp,v}$) = 0.0 $\mu V$

	Map	*ACC* (%)									
Fibrosis		Fixed electrode-to-tissue distance (FD)					Variable electrode-to-tissue distance (VD)				
marker		$\sigma _{v}$ (${\overline{V}}_{pp,v}$) $\mu V$					$\sigma _{v}$ (${\overline{V}}_{pp,v}$) $\mu V$				
		0.0 (0.0)	5.8 (24.2)	11.6 (48.4)	23.2 (96.7)	46.4 (193.5)	0.0 (0.0)	5.8 (24.2)	11.6 (48.4)	23.2 (96.7)	46.4 (193.5)
u-EGM EIGDR $2\times 2$ clique	$\mathcal {R}$	74.0	74.7±0.7	77.0±0.9	81.1±1.2	83.3±1.6	73.2±1.0	74.1±1.0	76.4±1.1	80.9±1.2	83.3±1.6
	$\mathcal{R}^{\mathcal{A}}$	86.2	85.2±0.9	85.7±1.2	87.2±1.3	86.3±1.6	85.0±0.8	84.5±1.0	85.4±1.2	87.1±1.3	86.2±1.6
	$\Delta \mathcal{R}^{\mathcal{A}}$	76.8	79.8±1.0	82.7±1.3	84.4±1.5	79.4±1.8	75.5±1.0	78.9±1.2	82.0±1.3	83.7±1.6	78.7±1.9
u-EGM EIGDR $3\times 3$ clique	$\mathcal {R}$	78.4	78.4±0.2	78.5±0.4	82.7±1.4	87.9±2.1	78.1±0.4	78.1±0.4	78.4±0.6	82.6±1.4	87.9±2.1
	$\mathcal{R}^{\mathcal{A}}$	92.1	91.8±1.2	92.2±1.4	94.0±1.4	94.2±1.6	92.3±0.8	91.9±1.2	92.3±1.4	94.0±1.4	94.2±1.6
	$\Delta \mathcal{R}^{\mathcal{A}}$	93.1	91.8±1.3	91.4±1.6	91.2±1.8	84.0±2.5	93.0±0.9	91.6±1.4	91.2±1.7	90.8±1.9	83.6±2.6
b-EGM amplitude	$V^{b\text {-}x}$	68.7	68.8±0.1	68.8±0.1	68.8±0.1	68.8±0.2	68.7±0.0	68.8±0.1	68.8±0.2	68.8±0.2	68.9±0.2
	$V^{b\text {-}y}$	90.8	90.9±0.3	90.9±0.4	88.7±0.7	82.5±1.0	86.8±1.0	86.9±1.0	87.3±1.0	86.5±1.0	81.6±1.1
	$V^{b\text {-}m}$	96.2	96.2±0.3	96.1±0.4	93.4±0.7	86.9±1.1	92.5±1.1	92.6±1.1	92.8±1.0	91.6±1.0	86.1±1.2

**Table 4:** Thresholds corresponding to the *ACC* values reported in Table 3, presented as mean ± standard deviation except for fixed electrode-to-noise distance and $\sigma _{v}$ ($\overline{V}_{pp,v}$) = 0.0 $\mu V$

	Map	Threshold									
Fibrosis		Fix electrode-to-tissue distance (FD)					Variable electrode-to-tissue distance (VD)				
marker		$\sigma _{v}$ (${\overline{V}}_{pp,v}$) $\mu V$					$\sigma _{v}$ (${\overline{V}}_{pp,v}$) $\mu V$				
		0.0 (0.0)	5.8 (24.2)	11.6 (48.4)	23.2 (96.7)	46.4 (193.5)	0.0 (0.0)	5.8 (24.2)	11.6 (48.4)	23.2 (96.7)	46.4 (193.5)
u-EGM EIGDR $2\times 2$ clique	$\mathcal {R}$	7.5	7.3±0.1	6.7±0.2	5.1±0.2	2.7±0.1	7.8±0.5	7.5±0.4	6.8±0.2	5.1±0.2	2.7±0.1
	$\mathcal{R}^{\mathcal{A}}$	86.8	69.1±3.3	39.3±2.1	14.5±0.7	4.3±0.2	87.3±2.9	67.7±3.4	38.7±2.1	14.4±0.7	4.3±0.2
	$\Delta \mathcal{R}^{\mathcal{A}}$	9.1	7.5±0.3	4.9±0.2	2.5±0.1	1.5±0.0	9.1±0.7	7.4±0.3	4.9±0.2	2.5±0.1	1.5±0.0
u-EGM EIGDR $3\times 3$ clique	$\mathcal {R}$	4.7	4.7±0.1	4.2±0.3	2.8±0.1	1.7±0.1	4.7±0.1	4.6±0.1	4.1±0.4	2.8±0.2	1.7±0.1
	$\mathcal{R}^{\mathcal{A}}$	36.9	32.1±1.0	23.1±0.8	10.7±0.4	3.5±0.1	36.1±0.6	31.8±1.0	22.9±0.8	10.7±0.4	3.5±0.1
	$\Delta \mathcal{R}^{\mathcal{A}}$	9.8	8.6±0.2	6.4±0.1	3.5±0.1	1.9±0.0	9.7±0.2	8.6±0.2	6.4±0.1	3.5±0.1	1.9±0.0
b-EGM amplitude (mV)	$V^{b\text {-}x}$	0.01	0.02±0.00	0.03±0.01	0.05±0.02	0.11±0.05	0.01±0.00	0.02±0.01	0.04±0.01	0.07±0.02	0.12±0.05
	$V^{b\text {-}y}$	0.76	0.76±0.00	0.76±0.00	0.79±0.01	0.84±0.02	0.76±0.01	0.76±0.01	0.77±0.01	0.79±0.01	0.84±0.02
	$V^{b\text {-}m}$	0.76	0.76±0.00	0.76±0.00	0.79±0.01	0.86±0.02	0.76±0.01	0.76±0.01	0.77±0.01	0.79±0.01	0.86±0.02

**Table 5:** Median values of EIGDR indices ($\mathcal {R}$,$\mathcal{R}^{\mathcal{A}}$and$\Delta \mathcal {R}^{\mathcal{A}}$) computed over the six cliques considered for the PentaRay^®^ catheter, median ($V^{b}$) and maximum ($V^{b\text {-}m}$) bipolar amplitude computed over the five innermost electrode pairs along the splines of the catheter, at different mapping points, taken at fibrotic (F) and non-fibrotic (NF) areas, respectively. Median and interquartile range (IQR) were also performed among F and NF points, separately

	# Catheter site	$\mathcal {R}$	$\mathcal{R}^{\mathcal{A}}$	$\Delta \mathcal{R}^{\mathcal{A}}$	$V^{b}$(mV)	$V^{b\text {-}m}$(mV)
F	1	3.42	4.79	1.04	0.04	0.60
	2	6.18	6.18	1.00	0.90	4.46
	3	2.45	4.75	1.95	1.65	3.61
	4	3.25	8.03	2.18	0.10	2.31
	5	1.47	5.35	3.03	1.46	1.63
	6	2.33	6.46	4.22	1.35	1.42
	7	2.22	2.24	1.02	0.16	0.30
	8	2.42	9.52	4.30	0.39	1.53
	9	2.18	7.24	4.17	1.31	3.06
	10	2.19	8.05	4.31	1.30	1.43
	11	3.40	9.92	3.06	0.25	0.50
	12	1.77	16.2	9.83	1.91	3.52
	13	1.56	2.56	1.57	1.01	2.00
	14	2.60	13.4	3.94	0.44	0.51
	15	0.91	2.27	1.83	0.84	3.73
	16	1.05	1.29	1.38	0.45	1.20
	17	1.18	4.90	4.57	1.46	1.89
	18	6.07	6.31	1.00	0.06	0.09
	19	1.80	3.70	3.00	0.37	5.04
	median/IQR	2.22/1.47	6.18/4.08	3.00/2.78	0.84/1.05	1.63/2.66
NF	1	4.54	8.08	2.44	1.42	2.06
	2	1.47	15.4	12.4	3.45	7.93
	3	2.33	6.46	2.61	1.15	1.72
	4	3.63	7.33	1.81	0.22	0.80
	5	2.06	4.62	1.66	1.36	4.51
	6	2.29	7.51	3.29	0.99	2.51
	7	1.51	16.5	9.97	6.90	10.2
	8	1.92	9.69	5.03	0.24	1.43
	9	1.65	6.51	3.14	0.88	1.39
	10	2.69	14.1	4.90	0.46	0.84
	11	2.45	4.75	1.95	1.65	3.61
	12	2.70	5.10	1.86	0.86	2.03
	13	2.95	6.29	1.75	0.14	0.39
	14	4.31	16.5	2.96	0.21	1.54
	15	2.19	7.61	3.54	0.25	1.36
	16	3.17	6.72	1.76	1.10	1.78
	17	2.72	6.11	1.80	1.05	4.02
	18	2.23	12.5	4.03	0.67	2.20
	19	2.61	5.89	2.22	0.27	1.20
	median/IQR	2.45/0.80	7.33/5.62	2.61/2.09	0.88/1.05	1.72/2.09
	*p*-value*	0.17	0.03	0.26	0.35	0.32

* refers to the comparison of markers between F and NF areas

**Table 6:** Median and interquartile range (IQR) of the EIGDR indices ($\mathcal {R}$, $\mathcal{R}^{\mathcal{A}}$ and $\Delta \mathcal{R}^{\mathcal{A}}$) computed individually on the six cliques of all catheter sites considered, and of bipolar amplitude values ($V^{b}$) computed individually on the five innermost electrode pairs of all catheter sites, at fibrotic (F) and non-fibrotic (NF) areas

		$\mathcal {R}$	$\mathcal{R}^{\mathcal{A}}$	$\Delta \mathcal{R}^{\mathcal{A}}$	$V^{b}$ (mV)
F	median/IQR	2.14/2.13	5.85/5.62	2.17/3.12	0.55/1.26
NF	median/IQR	2.43/2.34	7.42/6.74	2.67/3.25	0.80/1.24
	*p*-value*	0.08	0.0004	0.01	0.16

* refers to the comparison of markers between F and NF areas

## Conclusions

In this paper we demonstrated that mapping strategies based on the EIGDR method are able to discriminate fibrotic from non-fibrotic tissue. In simulation, they attain comparable performance to map obtained by combining the b-EGMs amplitudes along the two directions of the MEA for low noise levels, when assuming both fixed and variable distance between the electrode grid and the tissue. Nevertheless, they outperform bipolar maps when higher noise levels are added. Moreover, performance of $3\times 3$ electrode cliques outperforms the $2\times 2$ cliques one and fibrosis detection benefits from the previous time alignment of u-EGMs. With clinical data, EIGDR approach showed promising results in discriminating fibrotic and non-fibrotic mapping points, especially when u-EGMs are previously aligned in time. Both scenarios studied lead to choose $\mathcal{R}^{\mathcal{A}}$ as EIGDR biomarker for fibrosis discrimination.

## Data Availability

The raw data supporting the conclusions of this article will be made available by the authors, without undue reservation.

## Appendix

Table 7.

**Table 7:** List of acronyms and symbols

	Acronyms		Symbols
A	aligned	$I_{to}$	transient outward potassium current
ACC	maximum detection accuracy	$I_{CaL}$	L-Type calcium current
AF	atrial fibrillation	$I_{K1}$	inward rectifier potassium current
b-EGM	bipolar electrogram	$I_{Kur}$	ultrarapid outward potassium current
cAF	persistent atrial fibrillation	$I_{Ks}$	slow delayed rectifier potassium current
EAM	electroanatomical mapping	$\delta t$	constant time step in the monodomain formulation
EGM	electrogram	$\delta x$	spatial resolution in the monodomain formulation
EIGDR	eigenvalue dominance ratio	*L*	number of electrodes in the MEA
F	fibrotic	*d*	inter-electrode distance in MEA and PentaRay^®^ catheter
IIR	image intensity ratio	$\Psi$	MEA-to-tissue rotating angle
IQR	interquartile range	(*i*, *j*)	spatial coordinates for electrode location in the MEA
LA	left atrium	$u_{i,j}(n)$	u-EGM model
MEA	multi-electrode array	$\mu _{i,j}$	variable electrode-to-tissue distance in the MEA
MRI	magnetic resonance image	$\overline{\mu }$	mean of $\mu _{i,j}$
NA	non-aligned	$\sigma _{\mu }$	SD of $\mu _{i,j}$
NF	non-fibrotic	*N*	number of u-EGM samples
ROC	receiver operating characteristic	$\sigma _{v}$	SD of noise recording
SD	standard deviation	$\overline{V}_{pp,v}$	peak-to-peak amplitude of real noise recording
u-EGM	unipolar electrogram	*q*	index for noise recording realization
		$u^{q}_{i,j}(n)$	noisy u-EGM realization
		*K*	number of u-EGM signals in a clique
		$u_{k}(n)$	*k*-th u-EGM signal in the clique
		$\mathbf {u}_{k}$	$u_{k}(n)$ samples vector
		$\mathbf {U}$	u-EGMs samples matrix
		$\mathbf {R}_{u}$	intra-signal sample correlation matrix in the clique
		$\hat{ \mathbf {R}}_{u}$	intra-signal sample correlation matrix estimate in the clique
		$\lambda _{n}$	eigenvalue of $\hat{ \mathbf {R}}_{u}$
		$\mathcal {R}$	dominant-to-remaining eigenvalue ratio
		$u_{max}(n)$	highest peak-to-peak amplitude u-EGM in the clique
		$\bar{u}(n)$	average u-EGM in the clique
		*s*(*n*)	u-EGM activation signal component
		$E_{s}$	energy of *s*(*n*)
		$\tau _{k}$	delay of the *k*-th u-EGM with respect to a time reference within the clique
		$\beta ^{2}\sigma _{\tau }^{2}$	variance of $\tau _{k}$ ($\beta =$ 1 in non-fibrotic tissue; $\beta$ > 1 in fibrotic tissue)
		$\alpha _{k}$	u-EGM amplitude factor ($\alpha _{k}=1$ in non-fibrotic tissue; $\alpha _{k}<1$ in fibrotic tissue)
		$\overline{\alpha }$	mean of $\alpha _{k}$
		$\sigma _{\alpha }^{2}$	variance of $\alpha _{k}$
		$f_{k}(n)$	zero-mean fibrotic signal component in $u_{k}(n)$
		${\sigma _{f}^{2}}$	variance of $f_{k}(n)$
		$v_{k}(n)$	zero-mean Gaussian and white noise component at the *k*-th u-EGM
		${\sigma _{v}^{2}}$	variance of $v_{k}(n)$
		$\mathcal{R}^{\mathcal{A}}$	EIGDR with prior alignment and no fibrosis
		$s^{\prime }(n)$	first derivative of *s*(*n*)
		$s^{\prime \prime }(n)$	second derivative of *s*(*n*)
		$\mathbf {s}^{\prime }$	vector counterpart of $s^{\prime }(n)$
		$\mathbf {s}^{\prime \prime }$	vector counterpart of $s^{\prime \prime }(n)$
		$\mathcal{R}_{\mathcal{F}}$	EIGDR with no prior alignment and fibrosis
		$\mathbf {R}_{u}^{\bullet }$	inter-signal correlation matrix in the clique
		$\hat{\mathbf {R}}_{u}^{\bullet }$	inter-signal correlation matrix estimate in the clique
		$\lambda _{n}^{\bullet }$	eigenvalue of $\hat{\mathbf {R}}_{u}^{\bullet }$
		$\Delta {\mathcal{R}_{\mathcal{F}}}$	gain in EIGDR by healthy tissue again fibrotic one, in a clique
		$\Delta {\mathcal{R}^{\mathcal{A}}}$	gain in EIGDR produced by previous alignment in the clique
		$b_{i,j}^{x}(n)$	b-EGM along *x* direction of the MEA
		$b_{i,j}^{y}(n)$	b-EGM along *y* direction of the MEA
		${V_{i,j}^{b\text{- }x}}$	peak-to-peak amplitude of $b_{i,j}^{x}(n)$
		${V_{i,j}^{b\text{- }y}}$	peak-to-peak amplitude of $b_{i,j}^{y}(n)$
		${V_{i,j}^{b\text{- }m}}$	maximum between ${V_{i,j}^{b\text{- }x}}$ and ${V_{i,j}^{b\text{- }y}}$
		$V^{b}$	median value among all the bipolar amplitudes along the PentaRay^®^ catheter splines
		$V^{b\text {-}m}$	maximum value among all the bipolar amplitudes along the PentaRay^®^ catheter splines
		$\hat{\sigma }_{v}^{2}$	estimated noisy recording variance
		$\mathcal {T}$	threshold corresponding to *ACC* value
		$\Delta$	threshold offset from estimated EIGDR to control sensitivity to specificity performance
		$\hat{\Delta }$	estimated $\Delta$ from simulations
